# A Rapid and Complete Photodegradation of Doxycycline Using rGO@CuO Nanocomposite Under Visible and Direct Sunlight: Mechanistic Insights and Real-Time Applicability

**DOI:** 10.3390/nano15130953

**Published:** 2025-06-20

**Authors:** Panchraj Verma, Subrata Das, Shubham Raj, Raphaël Schneider

**Affiliations:** 1 Applied Chemistry Lab, Department of Chemistry, National Institute of Technology Patna, Bihar 800005, India; panchrajv.phd20.ch@nitp.ac.in (P.V.); shubhamr.phd22.ch@nitp.ac.in (S.R.); 2 LRGP, CNRS, Université de Lorraine, F-54000 Nancy, France; raphael.schneider@univ-lorraine.fr

**Keywords:** hydrothermal, graphene, visible light, complete degradation

## Abstract

In this study, a simple and efficient hydrothermal strategy was developed to modify reduced graphene oxide (rGO) with copper (II) oxide (CuO) nanoparticles by varying the weight ratio of rGO relative to CuO (rGO@CuO_1:1_, rGO@CuO_1:2_, and rGO@CuO_2:1_). The obtained materials were further characterized using analytical tools. Photocatalytic performance was assessed using adsorption–photocatalysis experiments under a household LED light source (10 W, λ > 400 nm), and the degree of degradation of doxycycline (DOX) was evaluated using UV-Vis spectrophotometer. The highest efficiency of 100% was achieved with a DOX concentration of 70 ppm, rGO@CuO_1:1_ dosage of 1 mg/mL, and pH 7 within 30 min of irradiation. The degradation kinetics followed the pseudo-first-order model (R^2^ ~0.99) and the Langmuir adsorption isotherm, indicating that DOX on the surface is governed by a dynamic equilibrium between adsorption and degradation rates. Furthermore, efficacy was tested using real water samples, and the recyclability of the catalyst was evaluated in up to five cycles.

## 1. Introduction

Doxycycline (DOX), a tetracycline-class antibiotic, exhibits a very broad spectrum of activity and is extensively used as medicine for both human and veterinary purposes due to its effectiveness against several bacterial infections [1]. Its clinical applications consist of the treatment of infections affecting the respiratory system and skin [2], certain sexually transmitted diseases [3], and malaria [4]. Although DOX offers significant medical benefits, its increasing presence as an environmental contaminant in aquatic ecosystems has raised considerable concern regarding its effects on both ecosystems and human health [5]. Pharmaceutical compounds often enter the environment through various industrial discharges [6], municipal waste [7], directly through patient excreta, solid waste disposal, hospital effluents, and sewage systems [8], as well as from livestock farming and aquaculture operations [9,10,11,12]. A significant proportion of antibiotics, estimated to be around 30–90%, are excreted from humans and animals through urine and feces either in their original form or as an active metabolite [13]. This mainly results from an incomplete absorption of these drugs within the gastrointestinal tract. Thus, antibiotics bypass conventional waste management systems, raising concerns about their environmental impacts [14].

DOX exhibits high stability and is significantly resilient to natural degradation processes, which enables this compound to persist in the environment for long periods [15], leading to its accumulation in water bodies. The production and consumption of DOX have increased significantly in recent years, as it has emerged as an alternative treatment for COVID-19 symptoms by mitigating excessive cytokine production and preventing lung damage [16]. The occurrence of DOX in water shows detrimental effects on aquatic organisms, disrupting microbial community growth [17] and facilitating bacterial resistance development by enabling mutations that allow bacteria to survive [18]. Antibiotic pollution disrupts the aquatic microorganisms that are essential for maintaining the ecological balance, promotes the emergence of resistant strains, and interferes with nutrient cycling and energy flow within aquatic ecosystems [19]. Moreover, the emergence of antibiotic resistance in bacteria poses a significant risk to human health, as these resistant strains can infiltrate the food chain or be transmitted to humans either through indirect or direct contact with contaminated water [20]. Considering these factors, the extensive adverse effects of DOX have prompted the development of various decontamination methods.

At present, numerous research efforts are focused on the development of advanced treatments capable of removing or degrading antibiotic residues from water. Several methods have been reported, including electrocoagulation [21], biodegradation [22], advanced oxidation [23], adsorption [24,25], and photodegradation techniques [26,27] for the removal of pollutants. For example, Zaidi et al. have shown the electrocoagulation-based removal of DOX using an electrochemical reactor [28]. He et al. reported the biotransformation of DOX using *Brevundimonas naejangsanensis* and *Sphingobacterium mizutaii* strains [29], while Spina-Cruz et al. reported the degradation of DOX using advanced oxidation processes [23]. However, significant drawbacks associated with these methods include high costs, need of sophisticated experimental setups, longer duration, incomplete removal, and the use of harsh oxidizers, which pose various health risks. Photocatalytic degradation offers a sustainable approach that provides several advantages, such as cost-effectiveness, recyclability, rapid onsite degradation, and durability, making it a more environmentally friendly and reliable approach. It primarily relies on the interaction of surface-active sites on the material to adsorb contaminants and their ability to generate charge carriers under irradiation, which facilitates the mineralization of the pollutants [30].

Reduced graphene oxide (rGO) stands out among numerous adsorbent materials as an efficient material for photocatalysis due to its surface functional groups (carboxylic acids, alcohols, epoxy, etc.), which facilitate pollutant adsorption, and its high electron mobility originating from the sp^2^ domain [31]. When combined with light-sensitive materials, its light absorption capacity and charge carrier separation ability are enhanced, which reduces the electron–hole recombination rate [32]. This boosts the production of active radicals that contribute to the breakdown of organic pollutants [33]. Numerous studies have established the application of rGO-based nanocomposites for photocatalytic applications. For example, Alwan et al. reported the photodegradation of Rhodamine B using visible light and rGO@TiO_2_ composite [34]. Similarly, Elumalai et al. reported the photodegradation of Rhodamine B and Methyl orange using rGO@ZnO composite [35], while Heng et al. synthesized a rGO@polyoxibinate photocatalyst and applied it for the photodegradation of tetracycline antibiotics [36]. The main advantage of using rGO-based nanocomposites originates from the high electron mobility due to the π-conjugated system. The extensive π conjugation and hydrogen bond formation further enhance their adsorption and photodegradation efficiency. Thus, the cost-effectiveness and reusability of rGO-based nanocomposites lead to the development of new heterogeneous photocatalysts.

Copper (II) Oxide (CuO) is a p-type semiconductor and it has the advantages of abundant Cu, low production cost, low toxicity, high chemical stability in aqueous medium, narrow band gap, and a high conductivity of 10^−4^ S/cm, which makes it a potential material to be used for photodegradation application [37,38]. Due to these properties, CuO has been extensively utilized as a photocatalyst, but due to its high recombination rate and lower charge transfer rate, it needs to be combined with other nanoparticles to form a nanocomposite, which overall increases its photodegradation efficiency [39,40]. Sakib et al. reported the photodegradation of methylene blue dye using the CuO/ZnO nanocomposite under solar irradiation [41]. Similarly, Ranjeh et al. used CuO/Li_3_BO_3_ for the photodegradation of dye acid violet 7 using an Osram lamp [42], while Raha et al. utilized a CuO/Mn_3_O_4_/ZnO nanocomposite for the photodegradation of Rabeprazole from water using a white LED lamp [43]. There are numerous similar studies where CuO-based nanocomposites were used as photocatalysts, and the main advantage of using CuO is its visible light activation [44].

Herein, a simple and environmentally friendly approach was developed for the synthesis of rGO@CuO nanocomposites. This method involves the surface modification of rGO with CuO to enhance its light absorption and promote the generation of photo-induced charge carriers, which contribute to the photodegradation of DOX from wastewater. Notably, we reported a rapid (30 min) and complete (100%) photocatalytic degradation of DOX using both a domestic white LED light source (10 W) and direct sunlight. These prominent features of our photocatalyst distinguish it from other conventional photocatalysts, which utilize costly light setups or photoreactors and require longer durations, thereby providing a novel aspect to our findings. Various parameters were also systematically investigated, including the dosage of the nanocomposite, the pH of the solution, the catalyst composition, and analyte concentrations, to establish the ideal conditions for the simultaneous adsorption and photodegradation of DOX. Our approach emphasizes the importance of the sustainability, efficiency, cost-effectiveness, and recyclability of the rGO@CuO catalyst, providing a practical strategy for eliminating pharmaceutical pollutants from contaminated water.

## 2. Materials and Methods

### 2.1. Chemicals and Reagents

Graphite powder (CAS:7782-42-5) was sourced from Merck Millipore, India. Sulfuric acid (H_2_SO_4_, 98% *v*/*v*) [CAS:7664-93-9], nitric acid (HNO_3_, 69–72%) [CAS:7697-37-2], hydrochloric acid (HCl, 35–38% *v*/*v*) [CAS:7647-01-0], and sodium hydroxide (NaOH, 97%) [CAS:1310-73-2] were purchased from CDH chemicals, India. Copper nitrate [CAS:10031-43-3] and hydrogen peroxide (H_2_O_2_, 30% *v*/*v*) [CAS:7722-84-1] were obtained from TCI and Fisher Scientific, respectively. Additionally, potassium permanganate (KMnO_4_) [CAS:7722-64-7] was acquired from FINAR chemicals. Isopropanol (IPA, C_3_H_8_O, 99%) [CAS:67-63-0], and potassium iodide (KI, 99.8%) [CAS:7681-11-0] were purchased from SRL Chemicals Pvt. Ltd., India. 1,4-Benzoquinone (BQ, C_6_H_4_O_2_, 98%) [CAS:106-51-4] was purchased from Sigma-Aldrich, India. Doxycycline powder (CAS RN: 24390-14-5) was obtained from TCI chemicals, while generic doxycycline hydrochloride (DOX) tablets were purchased from a local medical shop. Double-distilled water (DDW) was used throughout the experimental procedures.

### 2.2. Characterization

In this study, a series of analytical techniques were employed to thoroughly characterize the samples. UV-vis spectroscopy measurements were performed using a UV-Vis spectrophotometer (Evolution 300, Thermo Scientific, Waltham, MA, USA) over a wavelength range of 200 to 600 nm. Fourier-transform infrared (FT-IR, IRAffinity-1S, Shimadzu, Kyoto, Japan) spectra were recorded in the range of 4000 to 400 cm^−1^ and conducted in attenuated total reflectance (ATR). Powder X-ray diffraction (XRD, Smartlab, Rigaku Corporation, Tokyo, Japan) measurements were carried out using a Seifert X-ray diffractometer C-3000, covering a 2θ range of 5 to 80°, using Cu-Kα radiation at a voltage of 35 kV. Fluorescence emission spectra were recorded using a Fluoromax 4 (Horiba, Kyoto, Japan) spectrometer in the 360–560 nm range. High-resolution transmission electron microscopy (HR-TEM) imaging was conducted with a JEM 2100 (JEOL, Tokyo, Japan) operating at an acceleration voltage of 200 kV. XPS analysis was performed using a Scienta SES 200-2 (Gammadata, Uppsala, Sweden) spectrometer. Total organic carbon (TOC) estimation analysis was performed using the TOC-L series (Shimadzu, Kyoto, Japan).

### 2.3. Synthesis of GO, rGO, CuO, and rGO@CuO

The synthesis of graphene oxide (GO) was performed following a previously established methodology with minor modifications [45]. In brief, 1 g of graphite powder was added to a pre-stirred 200 mL acid solution composed of H_2_SO_4_ and H_3_PO_4_ (9:1 *v*/*v*). The solution temperature was maintained at approximately 10 °C beforehand, slowly adding 9 g of KMnO_4_, with careful temperature control to avoid exothermic reactions. Once the addition was complete, the mixture was stirred at a temperature of 55 °C for 12 h. Subsequently, the workup process involved the careful addition of ice-cold DDW along with 3 mL of 30% H_2_O_2_. The solution was then washed multiple times via centrifugation with DDW and ethanol; thereafter, GO was isolated by drying in a vacuum oven at 70 °C overnight.

**Scheme 1:**
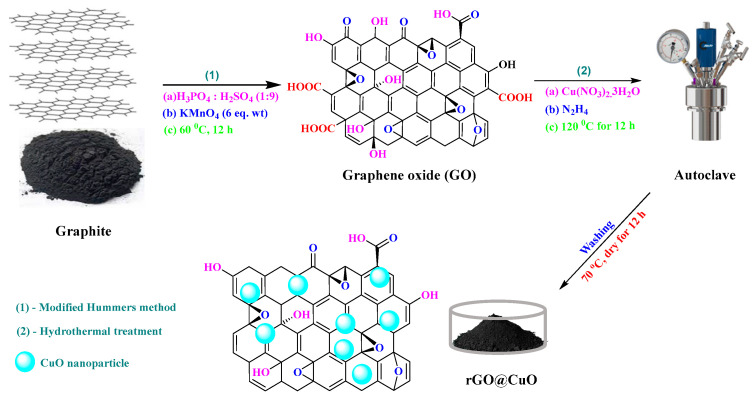
Stepwise hydrothermal synthesis of the rGO@CuO photocatalyst.

The synthesis of rGO@CuO_1:1_ was conducted in accordance with a previously established method with minor modifications [46]. Briefly, 100 mg of synthesized GO was dispersed in 60 mL of DDW using ultrasonication for 60 min. After complete dispersion, 100 mg of copper (II) nitrate trihydrate (Cu(NO_3_)_2_·3H_2_O) was slowly added to the GO solution and the mixture magnetically stirred for 1 h. The pH of the mixture was then adjusted to ~10 using 0.1M NaOH aqueous solution before transferring the solution to a Teflon-lined autoclave, which was heated at 120 °C for 12 h. The resultant black precipitate was isolated by multiple washing with DDW via centrifugation at 5000 rpm for 30 min. Finally, rGO@CuO_1:1_ was collected by drying overnight in a vacuum oven at 60 °C. Similarly, different ratios of rGO@CuO were prepared by varying weight equivalents of GO and Cu(NO_3_)_2_.3H_2_O and these were labeled as rGO@CuO_1:2_ and rGO@CuO_2:1_. The overall synthesis of the nanocomposite is depicted in Scheme 1. Further, the synthesis of rGO and CuO was carried out separately using the same experimental procedure described for rGO@CuO nanocomposite. For rGO, the synthesis was carried out in the absence of Cu(NO_3_)_2_·3H_2_O, while for CuO, the synthesis was performed in the absence of GO, resulting in the individual formation of rGO and CuO.

### 2.4. Photodegradation Studies

Batch adsorption–photodegradation studies were carried out using a 10 W Philips white LED light (λ > 400 nm). A 500 ppm stock solution was prepared using DOX tablets and subsequently diluted with DDW to obtain DOX concentrations ranging from 10 to 100 ppm. Photodegradation studies were conducted by varying the DOX concentration (10–100 ppm), nanocomposite dosage (0.5–1.5 mg/mL), solution pH (3–10) and the compositional ratio (1:1, 1:2, and 2:1) of the nanocomposite. For all experiments, the samples were placed in the dark for 1 h prior to photodegradation to establish the adsorption–desorption equilibrium. The solutions were then irradiated with visible light (1 × 10 W, λ > 400 nm) in a closed box fitted with a cooling fan and magnetically agitated at 200 rpm for uniform exposure while being positioned 10 cm away from the light source. At regular intervals of 5 min, 5 mL of the mixture was sampled and centrifuged to remove the photocatalyst. The residual DOX concentration was measured using a UV-vis spectrophotometer. Each experiment was performed in triplicate to ensure reliability.

The photodegradation efficiency and adsorption capacity of rGO@CuO for DOX were calculated using Equations (1) and (2) as per earlier published reports [47,48].

(1) $$\%\;\mathrm{photodegradation}\;\mathrm{efficiency}\;(\mathrm{Qe})=\frac{C_{0}-C_{t}}{C_{0}}*100$$

(2) $$\mathrm{Qt}=\frac{C_{0}-C_{e}}{M}*V$$

where C_0_ is the initial concentration of DOX, C_t_ is the DOX concentration remaining after photodegradation, C_e_ is the equilibrium DOX concentration, V is the volume of the reaction mixture in liters (L), M is the mass of the photocatalyst in grams (g), and Qt (mg/g) is the adsorption capacity of the photocatalyst.

## 3. Results and Discussion

### 3.1. Characterization of Photocatalysts

**Figure 1:**
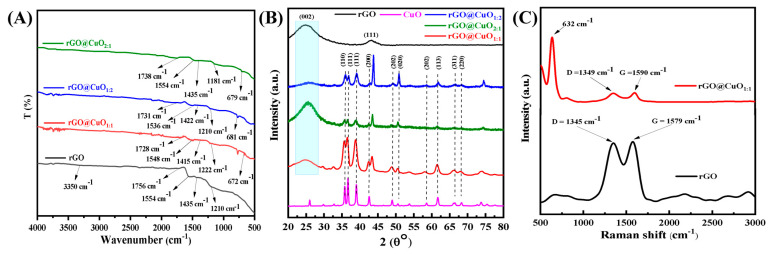
(**A**) FTIR spectra of rGO, rGO@CuO_1:1_, rGO@CuO_1:2_, rGO@CuO_2:1_; (**B**) XRD spectra of rGO, rGO@CuO_1:1_, rGO@CuO_1:2_, rGO@CuO_2:1_, CuO; (**C**) Raman spectra of rGO and rGO@CuO_1:1_.

The FT-IR spectra of rGO@CuO nanocomposites, with compositional ratios of 1:1, 1:2, and 2:1, were analyzed and compared with the FT-IR spectrum of rGO, as depicted in Figure 1A. The FT-IR spectrum of GO shown in Figure S1A shows a broad peak at ~3321 cm^−1^, corresponding to the O-H stretching vibration, as well as a sharp peak at ~1721 cm^−1^, attributed to the C=O stretching mode; these two peaks confirm the presence of OH and COOH groups [49]. Additionally, moderately intense peaks at 1623 cm^−1^ and 1073 cm^−1^ correspond to the C=C stretching of sp^2^ units and C-O-C stretching vibrations within GO, respectively [50]. For rGO, peaks of lower intensity are observed around 3350 cm^−1^ and 1756 cm^−1^, indicative of O-H and C=O stretching vibrations, respectively [51]. The peak at ~1560 cm^−1^ is attributed to C=C stretching and skeletal vibrations, while smaller peaks at 1156 cm^−1^ confirm the presence of the C-O-C stretching. The FT-IR spectrum of rGO shows a notable decline in peak intensity as compared to GO, confirming the reduction of GO into rGO [52]. Moreover, the FT-IR spectra of the rGO@CuO with various composition ratios, along with all the inherent peaks of rGO, show a consistent peak in the range of 700–760 cm^−1^ corresponding to the Cu-O stretching band associated with crystalline CuO, further confirming the formation of rGO@CuO composites [53].

For GO, rGO, CuO, and all the different composition ratios of the composites, lattice parameters (d-spacing) and crystallinity were analyzed using p-XRD. For GO, a distinct diffraction peak at 2θ = 10.38° corresponding to the (002) plane can be observed, which is indicative of its layered structure, while a broad peak around 2θ = 35° corresponding to (101) plane might originate from non-oxidized graphene sheets [54] (Figure S1B). For rGO, the peak at 24.97° corresponds to the (002) plane, indicative of residual edge and surface functional groups present in rGO, while a lower intensity peak at 43.01° denotes a turbostratic disordered arrangement within the rGO structure [55]. The diffractogram for the rGO@CuO_1:1_, rGO@CuO_1:2_, and rGO@CuO_2:1_ nanocomposites exhibits a broad peak located ~25° attributed to (002) plane of rGO and peaks at 35.76, 36.38, 38.85, 43.42, 48.78, 50.62, 58.56, 61.44, 66.07, and 68.21° attributed to the (110), (111), (111), (200), (202), (020), (202), (113), (311), and (220) planes, respectively, which matches the XRD pattern of monoclinic CuO. These results are in accordance with the earlier published result [56] (Figure 1B). Additionally, it can evidently be observed that the intensity of the peaks related to rGO and CuO varied with different composition ratios of the composites, confirming the binding of rGO and CuO in varying ratios. Also, the peak associated with rGO at 24.97° overshadowed the intense diffraction signal of CuO at 25.5°. The p-XRD analysis of rGO@CuO composite further confirmed the effective reduction of GO into rGO as the peak at 2θ = 10.38° is absent in the composite. Overall, the results indicated the effective functionalization of CuO onto the surface of rGO.

The Raman spectrum of GO is shown in Figure S1C, while those for rGO and the nanocomposite are presented in Figure 1C. For GO, two distinct peaks were observed at 1329 and 1558 cm^−1^, corresponding to the D and G bands, which arise due to the k-point phonon modes of A_1g_ symmetry and E_2g_ symmetry of sp^2^ carbon atoms in the graphene plane, respectively [57]. For rGO, D and G bands were observed at 1345 and 1579 cm^−1^, respectively. The increase in the ID/IG value from 0.91(GO) to 0.98 (rGO) specified an increase in defects due to partial restoration of the sp^2^ domain [58]. In the case of rGO@CuO_1:1_, these bands exhibit a red shift, appearing at 1349 cm^−1^ and 1590 cm^−1^ for the D and G bands, respectively, along with an additional peak at 632 cm^−1^ that corresponds to the B_2g_ symmetric vibrational mode of the Cu-O bond in the monoclinic CuO [59]. The shifts in the peak positions provide vital evidence for the successful synthesis of rGO@CuO_1:1_ nanocomposite [60].

**Figure 2:**
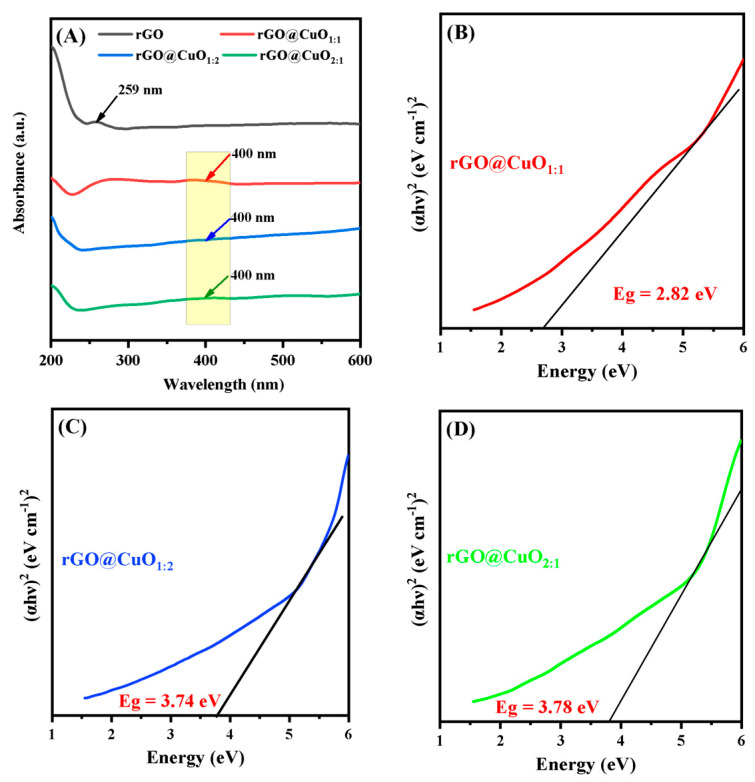
(**A**) UV-vis spectrum of rGO, rGO@CuO_1:1_, rGO@CuO_2:1_, and rGO@CuO_1:2_; (**B**–**D**) band gap calculations using Tauc plot for rGO@CuO_1:1_, rGO@CuO_1:2_, and rGO@CuO_2:1_.

The analysis of the UV-vis spectra of rGO and its nanocomposites at different composition ratios is illustrated in Figure 2A. The UV-visible spectrum of GO (Figure S2A) displays a prominent peak at 228 nm, associated with the specific π-π* electronic transition from sp^2^ centers. Additionally, a shoulder peak of ~300 nm is observed, which is associated with n-π* transition within the material [61]. The bandgap for GO calculated using the Tauc plot was 3.4 eV. For rGO, a broad absorption band observed around 259 nm is attributed to π-π* transition, while a red shift in this peak indicates the partial restoration of sp^2^-hybridized carbons. Additionally, the vanishing of the n-π* transition band suggests a significant reduction in the surface functional groups [62]. The bandgap calculated for rGO is 2.99 eV (inset of Figure S2B). For the nanocomposites, a newly formed broad absorption band centered around 400 nm, along with a low-intensity peak at 250 nm, was observed, which suggests the co-existence of rGO and CuO. The calculated band gaps for the rGO@CuO_1:1,_ rGO@CuO_1:2,_ and rGO@CuO_2:1_ nanocomposites are 2.82, 3.74, and 3.78 eV, respectively (Figure 2B–D). rGO@CuO_1:1_ exhibits the lowest bandgap, at 2.82 eV, making it highly suitable for visible light photodegradation.

The photoluminescence (PL) intensity of rGO and rGO@CuO nanocomposites was analyzed to evaluate the charge carrier recombination rate. The PL intensity was found to be maximum for rGO but decreased significantly for the nanocomposites, with the emission peak centered around 438 nm. For the nanocomposites, a reduction in PL intensity indicated the transfer of photogenerated electrons to rGO sheets, which effectively reduces the recombination rate. The rGO@CuO_1:1_ catalyst exhibited the lowest PL intensity (Figure S2C). These results led us to focus on the rGO@CuO_1:1_ composite for the study of DOX degradation.

**Figure 3:**
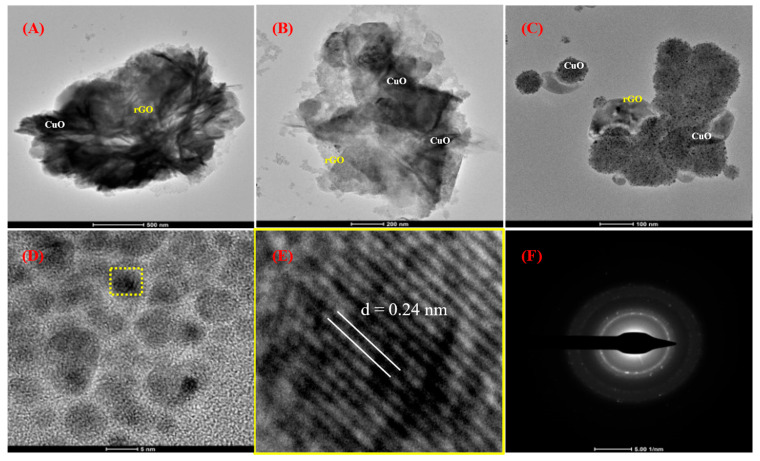
HR-TEM images of rGO@CuO_1:1_ nanocomposite at (**A**) 500 nm scale, (**B**) 200 nm scale, (**C**) 100 nm scale, (**D**) 5 nm scale; (**E**) zoomed-in image showing lattice fringes and (**F**) SAED pattern.

The HR-TEM images of the rGO@CuO_1:1_ nanocomposite at various magnifications, along with the selected area electron diffraction (SAED) pattern, are shown in Figure 3A–F, and those for rGO, CuO, rGO@CuO_1:2_, and rGO@CuO_2:1_ are shown in Figure S3A–H, Supporting Information. For rGO, a wrinkled translucent sheet-like morphology can be seen, and for CuO, similar sheets are observed, but with more darker spots. HR-TEM images of rGO@CuO_1:1_ display the uniform deposition of CuO onto the rGO surface (Figure 3A,B). At a higher magnification (Figure 3C), the HR-TEM image distinctly displays individual CuO nanoparticles as black spots dispersed uniformly across the rGO sheets, which confirms the creation of a heterojunction between rGO and CuO. Figure 3D shows the lattice fringes on the darker spots with a calculated d spacing of 0.24 nm (Figure 3E), which corresponds to the (111) plane of crystalline monoclinic CuO [63], further corroborating the p-XRD results. Further, the HRTEM images of rGO@CuO_1:2_ and rGO@CuO_2:1_ shows a morphology similar to that of rGO@CuO_1:_, but with an uneven distribution of rGO and CuO due to their unequal composition. The SAED pattern of rGO@CuO_1:1_further confirms the crystalline nature of the nanocomposite (Figure 3F). Moreover, EDX analysis was conducted for all three compositions to confirm the intended distribution of rGO and CuO, as shown in Figure S4, Supporting Information. This result successfully verifies the incorporation of rGO and CuO in the desired ratio, aligned with the synthesis design.

Earlier studies on DOX photodegradation have reported on photocatalysts with diverse structures and morphological characteristics, often incorporating multiple metal centers to enhance their photocatalytic efficiency [64]. In contrast to this, we report on a single metal-centered rGO@CuO nanocomposite for DOX photodegradation, demonstrating its unique structural and morphological features, which contribute to its photocatalytic performance.

**Figure 4:**
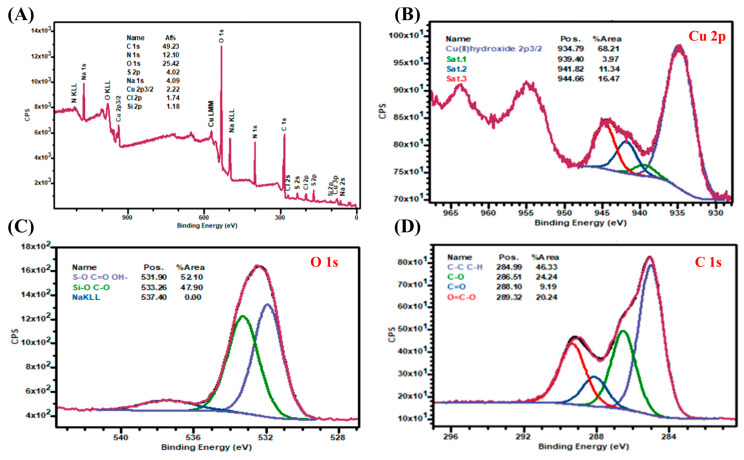
XPS spectra of the rGO@CuO_1:1_ nanocomposite (**A**) survey spectrum. High-resolution spectra of (**B**) Cu 2p, (**C**) O 1s, and (**D**) C 1s.

The elemental composition of the rGO@CuO_1:1_ nanocomposite was analyzed using XPS analysis (Figure 4). An overview of the XPS survey spectrum confirms the presence of C, O, and Cu elements in the rGO@CuO_1:1_ nanocomposite, indicating a successful incorporation of CuO onto the rGO framework (Figure 4A). The deconvoluted spectrum of Cu 2p depicted in Figure 4B shows two peaks corresponding to Cu 2p_3/2_ and Cu 2p_1/2_ at 934.79 eV and 954.23 eV, respectively. Additionally, the presence of three characteristic shake-up satellite peaks at 939.40, 941.82, and 944. 66 eV provides strong evidence for the presence of Cu in the +2 oxidation state [65,66]. The O 1s deconvoluted spectrum displays two peaks at 531.90 eV and 533.26 eV (Figure 4C), which can be attributed to lattice oxygen C=O, OH, and oxygen from C–O groups, respectively, with a decrease in residual oxygen-containing functional groups on the rGO surfaces [67]. The typical C 1s spectrum is deconvoluted into four sub-peaks located at 284.99 eV (C–C, C–H), 286.51 eV (C–O), 288.10 eV (C=O), and 289.32 eV (O=C–O), corresponding to rGO [65,68]. XPS results confirm the successful association of rGO with CuO.

### 3.2. DOX Adsorption–Photodegradation Studies

#### 3.2.1. Effect of DOX Concentration and rGO@CuO Compositional Ratio

**Figure 5:**
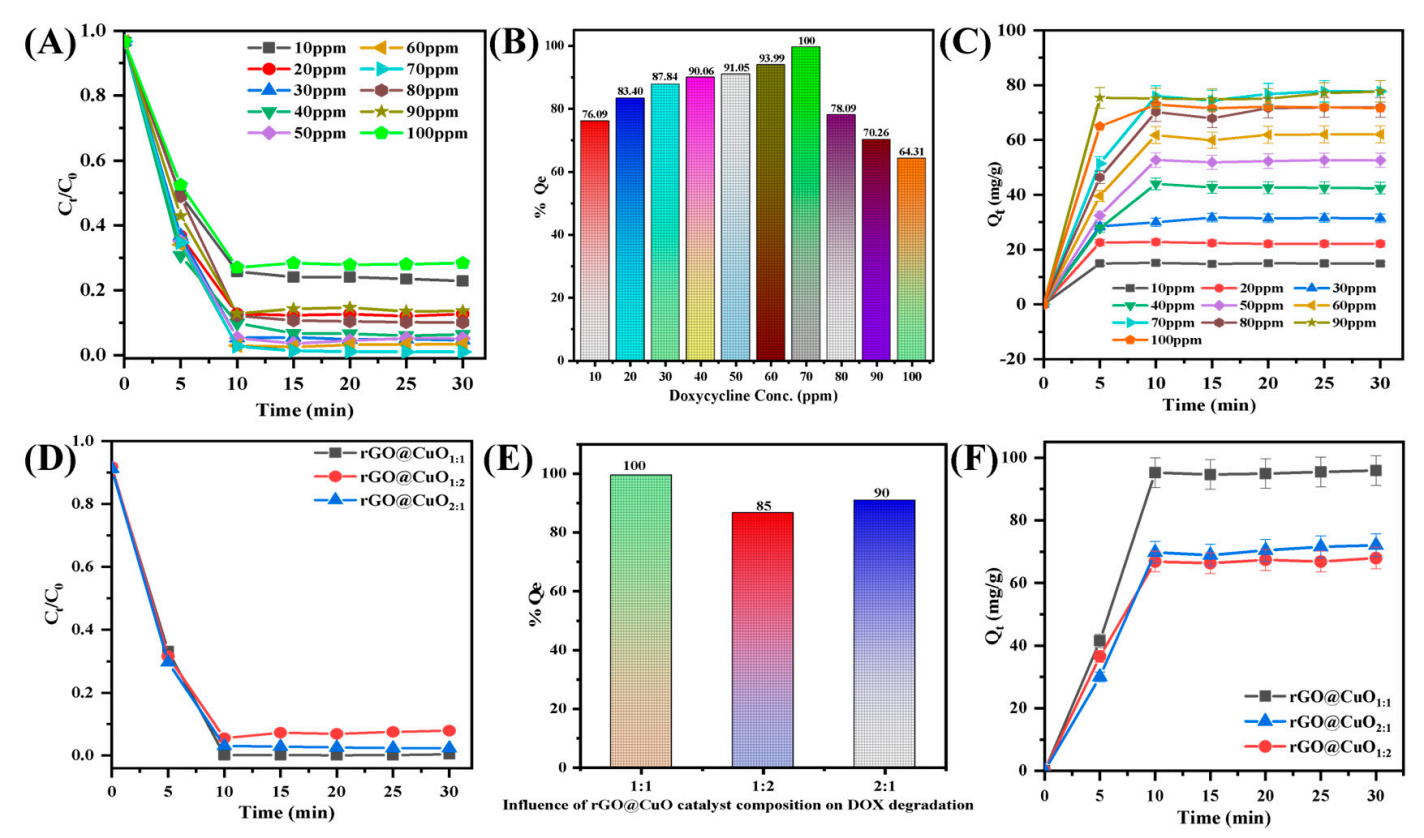
(**A**) Influence of DOX concentration on visible light photodegradation; (**B**) degradation efficiency (Qe) at varying DOX concentrations; (**C**) adsorption capacity (Qt) at different DOX concentrations (experimental conditions: rGO@CuO_1:1_, 1 mg/mL, neutral pH); (**D**) effect of rGO@CuO catalyst composition on DOX degradation; (**E**) degradation efficiency for different catalyst ratios; (**F**) adsorption capacity (Qt) of different catalyst compositions (experimental condition: DOX 70 ppm, catalyst dose 1 mg/mL, neutral pH).

The photocatalytic degradation of DOX under visible light irradiation using rGO@CuO catalysts was evaluated. The time needed to achieve an adsorption–desorption equilibrium in the dark prior to irradiation was carefully assessed. The UV-vis absorption spectra of DOX (70 ppm) were compared to the spectra of solution (DOX 70 ppm + rGO@CuO) kept under dark conditions. As shown in Figure S5, the adsorption–desorption equilibrium was achieved within 60 min. Preliminary photocatalytic tests evaluated the light-driven degradation of DOX (10–100 ppm) at pH 7, utilizing the rGO@CuO_1:1_ catalyst for performance evaluation and reaction efficiency assessment. To evaluate the effectiveness of photocatalysis, time-dependent changes in the maximum absorption at 360 nm (λ_max_) were recorded. The Qt values achieved with DOX at different concentrations ranging from 10 to 100 ppm are as follows: 14.89, 22.94, 31.40, 42.56, 52.59, 62.04, 77.06, 71.93, 77.72, and 71.60 mg/g, with corresponding degradation efficiencies (Qe) of 76.09, 78.29, 80.40, 88.18, 90.60, 91.25, 100, 80.80, 78.26, and 64.31% (Figure 5A–C and Figure S6). Maximum degradation occurred with 70 ppm DOX within 30 min of irradiation. Photodegradation efficiency initially increases gradually until 70 ppm and then tends to decline rapidly, which may be attributed to the saturation of the nanocomposite surface by DOX molecules [69]. These findings indicate that a DOX concentration of 70 ppm exhibits maximum Qe and Qt values, surpassing previously published reports. For instance, Yan et al. reported a maximum efficiency of ~95% for a DOX concentration of 15 ppm [70], while Wang et al. reported a maximum efficiency of ~89% for a 10 ppm DOX concentration [71]. Hereafter, a 70 ppm DOX concentration was consistently maintained while optimizing other parameters throughout the study.

Further, the influence of the rGO-to-CuO weight ratio in the rGO@CuO catalyst on DOX degradation was investigated (namely, the rGO@CuO_1:1_, rGO@CuO_1:2,_ and rGO@CuO_2:1_ catalysts were examined). All other parameters—DOX concentration, catalyst dosage, and pH—were fixed at 70 ppm, 1 mg/mL, and 7, respectively, throughout this optimization. The Qe values were 99.48, 86.65, and 90.97% for rGO@CuO_1:1_, rGO@CuO_2:1_, and rGO@CuO_1:2_, respectively (Figure 5E). Changing the weight ratio of pristine components led to a change in degradation efficiency. A decrease in CuO content (rGO@CuO_2:1_) reduced the light absorption ability despite the increased concentration of adsorption sites on rGO. This reduced light absorption diminished charge carrier generation, ultimately lowering the degradation rate and decreasing Qe [72]. Similarly, a decrease in the rGO content (rGO@CuO_1:2_) increased electron–hole pair recombination, which led to a decrease in photocatalytic activity, as confirmed by PL analysis. Earlier published results also show that varying the weight ratio of the pristine components affects the efficiency of the composite [73,74]. The UV-Vis absorption spectra during DOX degradation are illustrated in Figure S7. The quantified values of Qt follow the same trend, at 76.93, 70.96, and 67.96 mg/g for rGO@CuO_1:1_, rGO@CuO_2:1_, and rGO@CuO_1:2_, respectively (Figure 5F). These findings suggest that an optimal, i.e., equal, rGO-to-CuO weight ratio is required to maximize DOX degradation.

#### 3.2.2. Influence of rGO@CuO_1:1_ Catalyst Dosage and pH on DOX Photodegradation

**Figure 6:**
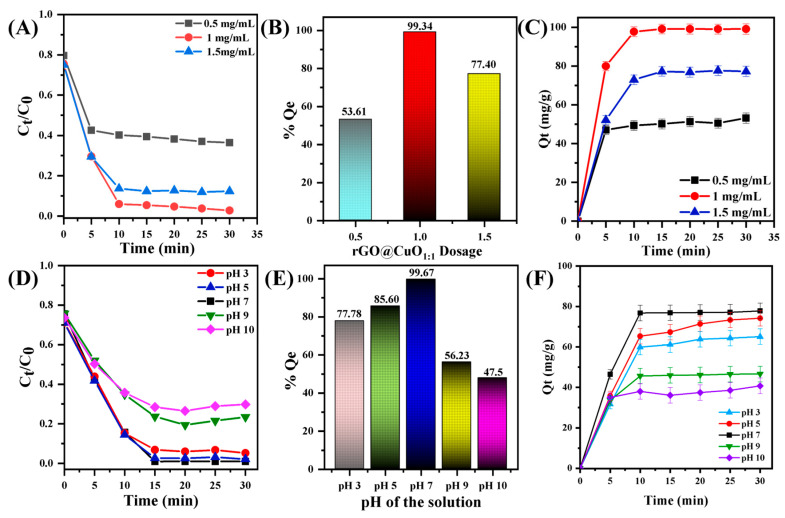
(**A**) Influence of the rGO@CuO_1:1_ catalyst loading on DOX degradation under visible light; (**B**) degradation efficiency; (**C**) influence of rGO@CuO_1:1_ catalyst dosage on adsorption capacity (reaction conditions: DOX 70 ppm, rGO@CuO_1:1_, and neutral pH); (**D**) effect of pH on degradation efficiency; (**E**) degradation efficiency (Qe); (**F**) adsorption capacity (Qt) at different pH (experimental conditions: DOX 70 ppm, rGO@CuO_1:1_ dosage of 1 mg/mL).

The effect of varying the dosage of the catalyst on the photodegradation of DOX was also investigated. For this, other parameters were maintained (70 ppm DOX, use of the rGO@CuO_1:1_ catalyst, and pH of ~7), while the photocatalyst dosage was varied from 0.5 to 1.5 mg/mL. The observed values of Qt were 89.91, 76.82, and 40.90 mg/g, while Qe was 53.61, 99.34, and 77.40% for 0.5, 1, and 1.5 mg/mL, respectively, indicating that the degradation efficiency depends on the dosage of the rGO@CuO_1:1_ catalyst. With an increase in dosage up to 1 mg/mL, the degradation efficiency increased; however, beyond this point, a reduction in the values of both Qt and Qe was observed. This can be explained by the surface-driven photocatalytic process, where the initial increase in degradation rate corresponds to an increase in the number of active photocatalytic sites, leading to enhanced generation of oxidative radicals. However, further increasing the amount of the catalyst beyond the optimal range results in a filtering effect, where the available light is insufficient to activate all the photocatalyst particles [75]. As a result, the overall photocatalytic activity dropped. Additionally, the increase in catalyst dosage intensified the competition among the adsorption sites for the limited number of DOX molecules in the solution. Consequently, any additional increase in catalyst dosage led to a decline in Qt and Qe values for the degradation of DOX. Earlier studies have reported dosages of 1.5 mg/mL and 1.25 mg/mL for an efficient degradation of DOX, values higher than the dosage used in this work (1 mg/mL), thus confirming the superior efficiency of rGO@CuO_1:1_ [70,76]. Details are presented in Figure 6A–C, and time-dependent UV-vis absorption spectra are shown in Figure S8.

The influence of pH on the photodegradation of DOX was further evaluated, as the surface charge of rGO@CuO_1:1_ and the generation of reactive radicals are influenced by pH [77]. The study was conducted using the above-optimized parameters and varying the pH from 3 to 10. DOX is cationic, zwitterionic, and anionic under acidic, neutral, and basic pH conditions, respectively. In contrast, the surface of rGO@CuO_1:1_ becomes increasingly negatively charged at higher pH values, as confirmed by the pH-dependent Zeta potential measurements depicted in Figure S9. The degradation efficiency was weak under acidic conditions, highest at near-neutral pH, and then decreased at basic pH (Figure 6D–F). At acidic pH, coulombic repulsions between the positively charged photocatalyst surface and protonated DOX molecules led to a decrease in degradation efficiency. Further, at neutral pH, the zwitterionic form of DOX enhanced the adsorption onto the catalyst surface, resulting in the highest degradation efficiency. At basic pH, the degradation efficiency decreases due to electrostatic repulsion between the negatively charged photocatalyst and the anionic form of DOX. The rGO@CuO_1:1_ achieves the maximum efficiency at pH 7, which is more relevant to wastewater treatment, unlike earlier reports focusing on extreme pH values of 3 and 10 [70,78]. The time-dependent UV-vis spectra for different pH values are shown in Figure S10.

#### 3.2.3. Effect of Photocatalyst Components on DOX Degradation

**Figure 7:**
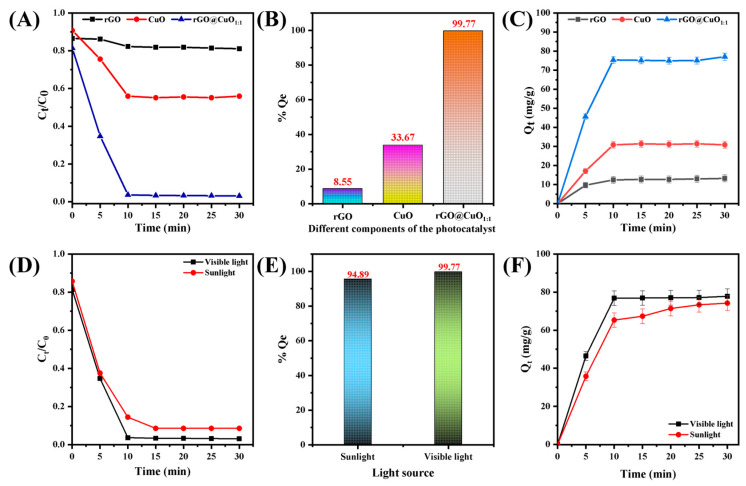
(**A**) Effect of catalyst components on DOX photodegradation; (**B**) degradation efficiency; (**C**) adsorption capacity for pristine components; (**D**) DOX photodegradation under natural sunlight; (**E**) degradation efficiency; (**F**) adsorption capacity in sunlight compared to artificial visible light (reaction conditions: DOX 70 ppm, rGO@CuO_1:1_ with a dosage of 1 mg/mL, neutral pH).

A control experiment was conducted with the pristine components (rGO and CuO) to evaluate the degradation efficiency in comparison to the nanocomposite. Using pure rGO, a degradation of 8% was observed, attributed to its limited light entrapment ability. Pure CuO shows a degradation efficiency of approximately 33%. However, when rGO and CuO were combined to form the nanocomposite, the degradation efficiency increased to ~100% within 30 min of irradiation (Figure 7A–C and Figure S11).

#### 3.2.4. Effect of Sunlight on DOX Degradation

The rGO@CuO_1:1_ catalyst was demonstrated to be highly effective for the photodegradation of DOX under visible light, but to assess its broader applicability, the photodegradation of DOX was further evaluated under natural sunlight. The experiments were performed under optimal conditions, i.e., a DOX concentration of 70 ppm, a 1 mg/mL dosage of the catalyst, and pH 7, using the rGO@CuO_1:1_ catalyst. The degradation result is closely aligned with visible light, which confirms the consistent catalytic performance of the catalyst. The calculated degradation efficiency (Qe) reached approximately 94.9% with a degradation extent (Qt) of 73.71 mg/g. These findings highlight the significant potential of rGO@CuO_1:1_ for environmental applications under natural sunlight without any major loss of activity (Figure 7D–F and Figure S12).

### 3.3. Real Sample and Reusability of the Photocatalyst

**Figure 8:**
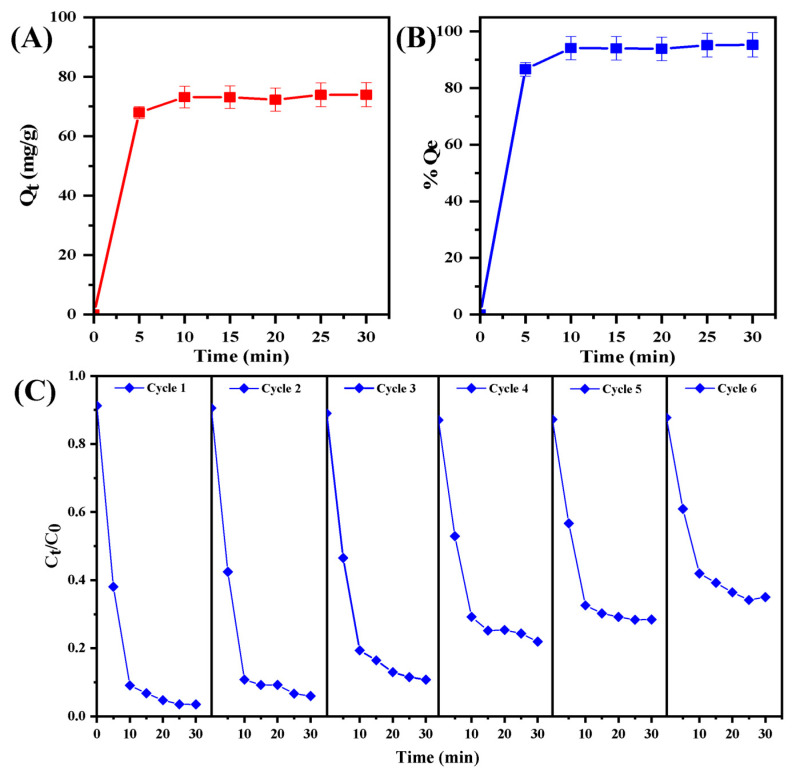
(**A**) Adsorption capacity and (**B**) degradation efficiency in degradation reaction using Ganga water; (**C**) reusability of the rGO@CuO_1:1_ photocatalyst.

The degradation of DOX was performed using a real water sample instead of DDW to evaluate the applicability of the photocatalyst. The real water sample was collected from the Ganga River at Gandhi Ghat, Mahendru region, Patna, Bihar. The total dissolved solid (TDS) and pH of the Ganga water were measured at 180 ppm and 7.2, respectively. Using a 0.45 µm syringe filter, the water sample was filtered to remove larger solid impurities and was then spiked with the optimized DOX concentration of 70 ppm. Photodegradation was carried out under previously optimized conditions, and the results are depicted in Figure 8A,B. The degradation of DOX in the real water sample was similar to that in distilled water, with calculated Qe and Qt values of 85% and 73 mg/g, respectively. These findings confirm the high efficiency of the photocatalyst even in water with high TDS.

The reusability of the photocatalyst was further assessed (Figure 8C). After each photodegradation cycle, the nanocomposite was recovered via centrifugation, washed with a water–ethanol mixture to remove any adsorbed impurities, and dried in a vacuum oven for 12 h before reuse. The photocatalyst retained a high degradation efficiency up to the fifth cycle (decrease to 55% after the sixth cycle). The decrease in degradation efficiency could be attributed to a partial abrasion of the photocatalyst caused by stirring, leading to a reduced photocatalytic activity [79]. To investigate any changes in the surface functional groups of the photocatalyst, FT-IR analysis was performed after cycles 1, 3, and 6 (Figure S13). The results indicated no significant changes, confirming the stability of the photocatalyst.

### 3.4. Isotherm Modeling and Kinetic Studies for DOX Degradation

Isotherm modeling studies were conducted to elucidate the nature of the degradation process. The mechanism of surface interactions during the photodegradation of DOX using the rGO@CuO_1:1_ catalyst was examined using three established theoretical models, i.e., the Langmuir isotherm, Freundlich isotherm, and Temkin isotherm. The Langmuir isotherm model was employed to investigate the monolayer adsorption of DOX onto rGO@CuO_1:1_. The linearized equations for the Langmuir model and *R_L_* mentioned in Equations (3) and (4) are as per previous studies [80].

(3) $$\frac{C_{e}}{Q_{e}}=\frac{1}{Q_{m}*K_{L}}+\frac{C_{e}}{Q_{m}}$$

(4) $$R_{L}=\frac{1}{\left(1+K_{L}*C_{o}\right)}$$

where Ce is the equilibrium concentration of DOX, Qe is the equilibrium adsorption capacity, *Q_m_* is the Langmuir constant, and *K_L_* is the Langmuir isotherm constant. *R_L_* is the separation factor, and its value determines the nature of adsorption, with *R_L_* = 0 indicating irreversible adsorption, *R_L_* > 1 unfavorable adsorption, and 0 < *R_L_* < 1 favorable adsorption [81].

The Freundlich isotherm model was used to describe the heterogeneous multilayer adsorption of DOX molecules onto the rGO@CuO_1:1_ surface during photodegradation, accounting for non-uniform adsorption heat. As per previous studies, the equation for the Freundlich isotherm model is shown in Equation (5) [80]:

(5) $$Log\;Q_{e}=Log\;K_{F}+\;\frac{1}{n}Log\;C_{e}$$

where *K_F_* and n represent the Freundlich constant and the adsorption constant, respectively.

The Temkin isotherm model was applied to explore the interaction between the photocatalyst and DOX molecules, proposing that adsorption is a function of uniformly distributed binding energy and that the molecular heat decreases during adsorption. The equation for the Temkin isotherm model is as per previous studies and shown in Equation (6) [82]:

(6) $$Q_{e}=B_{T}lnK_{t}+B_{T}lnC_{e}$$

where *B_T_* is the adsorption heat constant, and *K_t_* is the Temkin binding constant.

**Figure 9:**
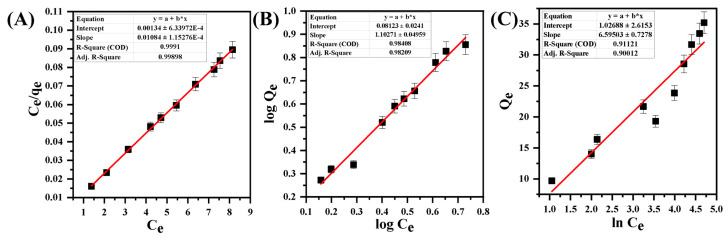
Isotherm modeling studies of DOX on rGO@CuO_1:1_: (**A**) Langmuir, (**B**) Freundlich, and (**C**) Temkin isotherm.

The results of the three-isotherm modeling study are illustrated in Figure 9A–C. The derived parameters are summarized in Table 1. Among the three models, the Langmuir isotherm exhibited the best fit, suggesting that DOX adsorption on rGO@CuO_1:1_ occurs on uniformly distributed surface sites, following a monolayer adsorption mechanism [83]. The Langmuir isotherm parameters were determined as Q_m_ = 89.68 mg^−1^, K_L_ = 0.065, and R^2^ ~0.99. Additionally, the calculated R_L_ value of 0.181 confirms the favorable nature of DOX adsorption on the rGO@CuO_1:1_ nanocomposite.

**Figure 10:**
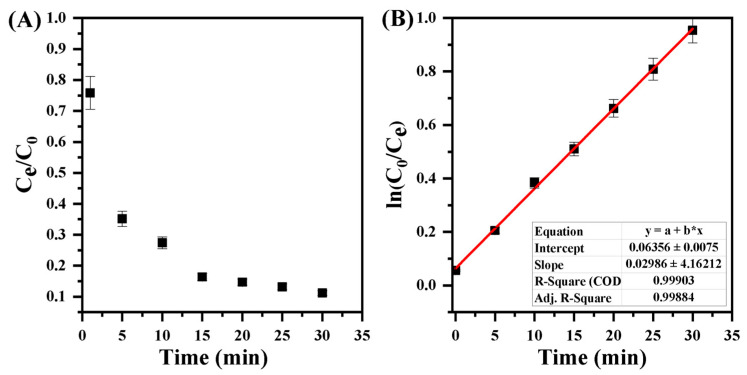
Plot for the pseudo-first-order kinetics study of the degradation of DOX by rGO@CuO_1:1_: (**A**) Ce/C_0_ vs. time (**B**) and ln (C_0_/Ce) vs. time.

Further, the photodegradation kinetics of DOX was analyzed using pseudo-first-order reaction kinetics as per earlier published reports [84]. The equation utilized for this analysis is given in Equation (7):

(7) $$C_{0}=C_{e}\left(1-e^{-k_{1}t}\right)$$

where *C*_0_ and *C_e_* represent the concentration of DOX for time t = 0 min and different irradiation times, respectively. *K_1_* represents the rate constant for pseudo-first-order kinetics and is determined using the slope of the graph ln(C_0_/C_e_) vs. time (min). The photodegradation kinetics of DOX at different concentrations were evaluated, and the results demonstrated high linearity with pseudo-first-order kinetics, as indicated by R^2^ ~0.99, shown in Figure 10. The calculated rate constant was *K*_1_ = 0.0296 min^−1^, highlighting a surface-mediated degradation process.

Additionally, the degradation efficiency of the rGO@CuO_1:1_ photocatalyst for DOX degradation was compared with that of earlier reported photocatalysts (Table 2). The findings reveal that the performance of rGO@CuO_1:1_ is either superior or comparable to that of previously reported photocatalysts in terms of DOX concentration, dosage, light source utilized, degradation time, or degradation efficiency for DOX degradation.

### 3.5. Mechanism of DOX Photodegradation

The mechanism of DOX photodegradation utilizing visible light and rGO@CuO_1:1_ was investigated. The generation and role of reactive species, including O_2_^•−^, OH^•^, and *h^+^*, were analyzed through scavenging experiments [95]. IPA, KI, and BQ were employed as scavengers for OH^•^, *h^+^*, and O_2_^•−^, respectively. The degradation efficiency dropped to 40.21, 28.56, and 15.43% when scavengers for *h^+^*, OH^•^, and O_2_^•−^ were used. This indicates that OH^•^ and O_2_^•−^ play a dominant role, while *h^+^* indirectly contributes to the degradation process. When scavengers for both OH^•^ and O_2_^•−^ were added, the degradation efficiency (Qe) dropped significantly to approximately 10.17%. These findings confirm that O_2_^•−^ and OH^•^ radicals are the primary contributors to the photodegradation of DOX (Figure S14).

**Scheme 2:**
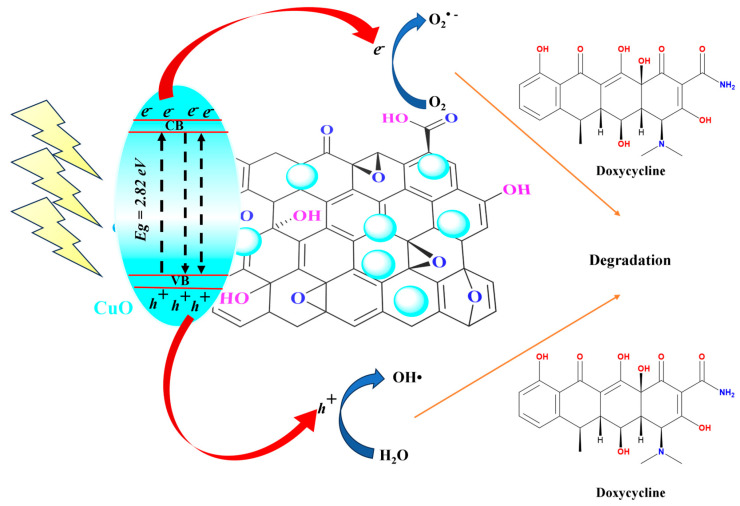
Illustration showing DOX photodegradation using rGO@CuO_1:1_.

Based on these observations, the following photodegradation mechanism can be proposed: Firstly, upon irradiation, the photocatalyst absorbs photons, which promotes e^-^ to the conduction band (CB), generating *h^+^* in the valence band (VB). The calculated values of E_CB_ and E_VB_ of CuO are −0.1 and 2.72 eV, respectively, while the Fermi level of rGO is −0.08 eV (see Supporting Information for calculations). The band edge difference between CuO and the Fermi level of rGO facilitates the transfer of photoexcited electrons to rGO, which effectively suppresses the recombination of *e^−^* *−* *h^+^* pair. The photoexcited electrons react with the adsorbed oxygen molecules to form O_2_^•−^ radicals, which drive the degradation of DOX. Simultaneously, h^+^ ions in the VB react with water molecules and oxidize them to generate OH^•^, which further contributes to the photodegradation process. The mineralization efficiency of rGO@CuO_1:1_ was evaluated using a TOC analyzer (detailed calculation in Supporting Information, Table S1). The analysis reveals that 68% of the initial organic carbon, i.e., DOX, was degraded into inorganic compounds primarily CO_2_. Based on this result, we can conclude that rGO@CuO_1:1_ can degrade a substantial amount of DOX into harmless benign products, highlighting its potential for wastewater treatment. The degradation pathway of DOX, based on the above findings, is represented through Equations (8)–(12) and shown in Scheme 2.

(8) $$\mathrm{rGO}@{\mathrm{CuO}}_{1:1}+h\upsilon \to \mathrm{CuO}\;(h^{+}+e^{-})$$

(9) $$\mathrm{CuO}\;(e^{-})\;\to \mathrm{rGO}(e^{-})$$

(10) $$e^{-}+O_{2}\to O_{2}^{•-}$$

(11) $$h^{+}+H_{2}O\to {\mathrm{OH}}^{•}+H^{+}$$

(12) $${\mathrm{OH}}^{•}+O_{2}^{•-}+\mathrm{DOX}\to \mathrm{Degradation}\mathrm{products}$$

## 4. Conclusions

We have shown the synthesis of the rGO@CuO_1:1_ photocatalyst via the hydrothermal method and achieved its exceptional performance as a photocatalyst for the photodegradation of DOX under visible light irradiation using a 10 W visible light source. PL analysis of the rGO@CuO_1:1_ nanocomposite confirmed a significant reduction in electron–hole pair recombination, allowing it for efficient photocatalytic activity. Under optimal conditions—a DOX concentration of 70 ppm, a catalyst loading of 1 mg/mL, and a pH level of 7—the rGO@CuO_1:1_ photocatalyst achieved complete 100% degradation of DOX within 30 min. Real-time applicability was validated using Ganga River water, where degradation efficiency remained consistent, with a minimal effect from the higher TDS level of 180 ppm. The photocatalyst showed high reusability over five cycles, with efficiency decreasing slightly due to abrasion. Kinetics and isotherm studies demonstrated pseudo-first-order kinetics and surface-mediated photodegradation with the Langmuir model, indicating monolayer adsorption and R^2^ ~ 0.99. Scavenging experiments identified OH^•^ and O_2_^•−^ as the key reactive species, while TOC analysis revealed 68% mineralization of DOX. The photocatalyst’s comparable performance under natural sunlight highlights its stability, scalability, and potential for industrial and environmental applications.

## Figures and Tables

**Table 1:** Calculated parameters for the three isotherm models.

Isotherm Model	Parameters		
Langmuir model	Q_m_	K_L_	R^2^
	89.686	0.065	0.99
Temkin model	B_T_	K_T_	R^2^
	0.046	1.047	0.919
Freundlich model	1/n	K_F_	R^2^
	110	84.23	0.98

**Table 2:** Comparison of DOX degradation efficiency.

Photocatalyst	DOX Conc. (ppm)	Dosage (mg/mL)	Light Source	Time (min)	Degradation (%)	Ref.
P25	25	0.1	300 W Xe lamp	80	26	[85]
ZnIn_2_S_4_/NiFe_2_O_4_/biochar	30	0.05	Visible light	120	66	[86]
MWCNTs/α-Bi_2_O_3_	10	0.05	150 W Xe lamp	120	91	[87]
rGO-Bi_2_Sn_2_O_7_-NiFe_2_O_4_	10	50	Visible light	270	97	[88]
BiOBr/FeWO_4_	N/A	1	Visible light 300 W	60	90	[73]
CoFe_2_O_4_/H_2_O_2_	20	60	N/A	60	90	[89]
5% g-C_3_N_4_@CeO_2_	10		Visible light 150 W	60	84	[90]
(NGQDs)-BiOI/MnNb_2_O_6_	10		250 W Xe lamp	60	87	[91]
Pd–TiO_2_	25		UVA (15 W)		100	[92]
Modified g-C_3_N_4_/MgZnA	10	50	Visible light	120	91	[93]
Fe/Pr_6_O_11_–MoO_3_@gC_3_N_4_ NCs	50	50	Visible light	220	98	[94]
rGO@CuO_1:1_	70	1	10 W LED	30	100	This work
rGO@CuO_1:1_	70	1	Sunlight	30	94	This work

## Data Availability

The data presented in this study are available upon request from the corresponding author.

## Supplementary Materials

The following supporting information can be downloaded at https://www.mdpi.com/article/10.3390/nano15130953/s1, Figure S1: FTIR, p-XRD, and Raman analysis of GO; Figure S2: UV-vis absorbance spectra of GO, rGO, and PL of nanocomposites; Figure S3: HRTEM analysis of pristine components and different compositional ratio of photocatalyst; Figure S4: EDX analysis of photocatalyst; Figure S5: Adsorption–desorption equilibrium analysis; Figure S6: Time-dependent UV-vis absorbance spectra for different concentrations of DOX; Figure S7: Time-dependent UV-vis absorbance spectra for different nanocomposite composition ratios; Figure S8: Time-dependent UV-vis absorbance spectra for different nanocomposite dosages; Figure S9: Zeta potential analysis; Figure S10: Time-dependent UV-vis absorbance spectra for different solution pH values; Figure S11: Time-dependent UV-vis absorbance spectra for pristine components of nanocomposite; Figure S12: Photodegradation study in sunlight; Figure S13: FTIR of recycled nanocomposite; Figure S14: Effect of scavengers on photodegradation; Table S1: TOC results before and after photodegradation. The authors have cited additional references in the Supplementary Materials [96,97].

## Abbreviations

The following abbreviations are used in this manuscript:

DOX	Doxycycline
GO	Graphene oxide
rGO	Reduced graphene oxide
DDW	Double-distilled water
FT-IR	Fourier-transform infrared
p-XRD	Powder X-ray diffraction
HRTEM	High-resolution transmission electron microscope
XPS	X-ray photoelectron spectroscopy
TOC	Total organic carbon
TDS	Total dissolved solid
CB	Conduction band
VB	Valence band

## References

[1] Berendsen B.J.A., Wegh R.S., Memelink J., Zuidema T., Stolker L.A.M. (2015). The Analysis of Animal Faeces as a Tool to Monitor Antibiotic Usage. Talanta.

[2] Navarro-Triviño F.J., Pérez-López I., Ruíz-Villaverde R. (2020). Doxycycline, an Antibiotic or an Anti-Inflammatory Agent? The Most Common Uses in Dermatology. Actas Dermosifiliogr..

[3] Molina J.M., Bercot B., Assoumou L., Rubenstein E., Algarte-Genin M., Pialoux G., Katlama C., Surgers L., Bébéar C., Dupin N. (2024). Doxycycline Prophylaxis and Meningococcal Group B Vaccine to Prevent Bacterial Sexually Transmitted Infections in France (ANRS 174 DOXYVAC): A Multicentre, Open-Label, Randomised Trial with a 2 × 2 Factorial Design. Lancet. Infect. Dis..

[4] Wilairatana P., Krudsood S., Treeprasertsuk S., Chalermrut K., Looareesuwan S. (2002). The Future Outlook of Antimalarial Drugs and Recent Work on the Treatment of Malaria. Arch. Med. Res..

[5] Grenni P., Ancona V., Caracciolo A.B. (2018). Ecological Effects of Antibiotics on Natural Ecosystems: A Review. Microchem. J..

[6] Sundararaman S., Kumar J.A., Deivasigamani P., Devarajan Y. (2022). Emerging Pharma Residue Contaminants: Occurrence, Monitoring, Risk and Fate Assessment–A Challenge to Water Resource Management. Sci. Total Environ..

[7] Wang C., Zhao Y., Liu S., Xiao Q., Liang W., Song Y. (2021). Contamination, Distribution, and Risk Assessment of Antibiotics in the Urban Surface Water of the Pearl River in Guangzhou, South China. Environ. Monit. Assess..

[8] Ali M., Wang W., Chaudhry N., Geng Y. (2017). Hospital Waste Management in Developing Countries: A Mini Review. Waste Manag. Res..

[9] Zhang Q.-Q., Ying G.-G., Pan C.-G., Liu Y.-S., Zhao J.-L. (2015). A Comprehensive Evaluation of Antibiotics Emission and Fate in the River Basins of China: Source Analysis, Multimedia Modelling, and Linkage to Bacterial Resistance. Environ. Sci. Technol..

[10] Qiao M., Ying G.G., Singer A.C., Zhu Y.G. (2018). Review of Antibiotic Resistance in China and Its Environment. Environ. Int..

[11] Kim H.Y., Lee I.S., Oh J.E. (2017). Human and Veterinary Pharmaceuticals in the Marine Environment Including Fish Farms in Korea. Sci. Total Environ..

[12] Carpenter L., Miller S., Flynn E., Choo J.M., Collins J., Shoubridge A.P., Gordon D., Lynn D.J., Whitehead C., Leong L.E.X. (2024). Exposure to Doxycycline Increases Risk of Carrying a Broad Range of Enteric Antimicrobial Resistance Determinants in an Elderly Cohort. J. Infect..

[13] Cheng D., Feng Y., Liu Y., Xue J., Li Z. (2019). Dynamics of Oxytetracycline, Sulfamerazine, and Ciprofloxacin and Related Antibiotic Resistance Genes during Swine Manure Composting. J. Environ. Manag..

[14] Bansal O.P. (2022). Antibiotics in the Environment: A Review. Int. J. Front. Biol. Pharm..

[15] Zaranyika M.F., Dzomba P., Kugara J. (2015). Speciation and Persistence of Doxycycline in the Aquatic Environment: Characterization in Terms of Steady State Kinetics. J. Environ. Sci. Health B.

[16] Malek A.E., Granwehr B.P., Kontoyiannis D.P. (2020). Doxycycline as a Potential Partner of COVID-19 Therapies. IDCases.

[17] Carrillo M.P., Sevilla M., Casado M., Piña B., López E.P., Matamoros V., Vila-Costa M., Barata C. (2023). Impact of the Antibiotic Doxycycline on the D. Magna Reproduction, Associated Microbiome and Antibiotic Resistance Genes in Treated Wastewater Conditions. Environ. Pollut..

[18] Sharma V.K., Johnson N., Cizmas L., McDonald T.J., Kim H. (2016). A Review of the Influence of Treatment Strategies on Antibiotic Resistant Bacteria and Antibiotic Resistance Genes. Chemosphere.

[19] Kraemer S.A., Ramachandran A., Perron G.G. (2019). Antibiotic Pollution in the Environment: From Microbial Ecology to Public Policy. Microorganisms.

[20] Ahmad I., Malak H.A., Abulreesh H.H. (2021). Environmental Antimicrobial Resistance and Its Drivers: A Potential Threat to Public Health. J. Glob. Antimicrob. Resist..

[21] Shahedi A., Darban A.K., Taghipour F., Jamshidi-Zanjani A. (2020). A Review on Industrial Wastewater Treatment via Electrocoagulation Processes. Curr. Opin. Electrochem..

[22] Wen X., Wang Y., Zou Y., Ma B., Wu Y. (2018). No Evidential Correlation between Veterinary Antibiotic Degradation Ability and Resistance Genes in Microorganisms during the Biodegradation of Doxycycline. Ecotoxicol. Environ. Saf..

[23] Spina-Cruz M., Maniero M.G., Guimarães J.R. (2019). Advanced Oxidation Processes on Doxycycline Degradation: Monitoring of Antimicrobial Activity and Toxicity. Environ. Sci. Pollut. Res..

[24] Zaidi S., Sivasankar V., Chaabane T., Alonzo V., Omine K., Maachi R., Darchen A., Prabhakaran M. (2019). Separate and Simultaneous Removal of Doxycycline and Oxytetracycline Antibiotics by Electro-Generated Adsorbents (EGAs). J. Environ. Chem. Eng..

[25] Kong Y., Wang L., Ge Y., Su H., Li Z. (2019). Lignin Xanthate Resin–Bentonite Clay Composite as a Highly Effective and Low-Cost Adsorbent for the Removal of Doxycycline Hydrochloride Antibiotic and Mercury Ions in Water. J. Hazard. Mater..

[26] Wu S., Lin Y., Hu Y.H. (2021). Strategies of Tuning Catalysts for Efficient Photodegradation of Antibiotics in Water Environments: A Review. J. Mater. Chem. A Mater..

[27] Wei L., Li H., Lu J. (2021). Algae-Induced Photodegradation of Antibiotics: A Review. Environ. Pollut..

[28] Zaidi S., Chaabane T., Sivasankar V., Darchen A., Maachi R., Msagati T.A.M. (2019). Electro-Coagulation Coupled Electro-Flotation Process: Feasible Choice in Doxycycline Removal from Pharmaceutical Effluents. Arab. J. Chem..

[29] He T., Bao J., Leng Y., Snow D., Kong S., Wang T., Li X. (2021). Biotransformation of Doxycycline by Brevundimonas Naejangsanensis and Sphingobacterium Mizutaii Strains. J. Hazard. Mater..

[30] Yu F., Tian F., Zou H., Ye Z., Peng C., Huang J., Zheng Y., Zhang Y., Yang Y., Wei X. (2021). ZnO/Biochar Nanocomposites via Solvent Free Ball Milling for Enhanced Adsorption and Photocatalytic Degradation of Methylene Blue. J. Hazard. Mater..

[31] Khan F., Khan M.S., Kamal S., Arshad M., Ahmad S.I., Nami S.A.A. (2020). Recent Advances in Graphene Oxide and Reduced Graphene Oxide Based Nanocomposites for the Photodegradation of Dyes. J. Mater. Chem. C Mater..

[32] Mondal A., Prabhakaran A., Gupta S., Subramanian V.R. (2021). Boosting Photocatalytic Activity Using Reduced Graphene Oxide (RGO)/Semiconductor Nanocomposites: Issues and Future Scope. ACS Omega.

[33] Albero J., Mateo D., García H. (2019). Graphene-Based Materials as Efficient Photocatalysts for Water Splitting. Molecules.

[34] Alwan S.H., Salem K.H., Alshamsi H.A. (2022). Visible Light-Driven Photocatalytic Degradation of Rhodamine B Dye onto TiO_2_/RGO Nanocomposites. Mater. Today Commun..

[35] Elumalai N., Prabhu S., Selvaraj M., Silambarasan A., Navaneethan M., Harish S., Ramu P., Ramesh R. (2022). Enhanced Photocatalytic Activity of ZnO Hexagonal Tube/r-GO Composite on Degradation of Organic Aqueous Pollutant and Study of Charge Transport Properties. Chemosphere.

[36] Heng S., Song Q., Liu S., Guo H., Pang J., Qu X., Bai Y., Li L., Dang D. (2021). Construction of 2D Polyoxoniobate/RGO Heterojunction Photocatalysts for the Enhanced Photodegradation of Tetracycline. Appl. Surf. Sci..

[37] Anandan S., Lee G.-J., Wu J.J. (2012). Sonochemical Synthesis of CuO Nanostructures with Different Morphology. Ultrason. Sonochem..

[38] Hsieh S.-H., Ting J.-M. (2018). Characterization and Photocatalytic Performance of Ternary Cu-Doped ZnO/Graphene Materials. Appl. Surf. Sci..

[39] Arai T., Yanagida M., Konishi Y., Iwasaki Y., Sugihara H., Sayama K. (2008). Promotion Effect of CuO Co-Catalyst on WO_3_-Catalyzed Photodegradation of Organic Substances. Catal. Commun..

[40] Mishra S.R., Ahmaruzzaman M. (2022). CuO and CuO-Based Nanocomposites: Synthesis and Applications in Environment and Energy. Sustain. Mater. Technol..

[41] Sakib A.A.M., Masum S.M., Hoinkis J., Islam R., Molla M.A.I. (2019). Synthesis of CuO/ZnO Nanocomposites and Their Application in Photodegradation of Toxic Textile Dye. J. Compos. Sci..

[42] Ranjeh M., Beshkar F., Amiri O., Salavati-Niasari M., Moayedi H. (2020). Pechini Sol-Gel Synthesis of Cu_2_O/Li_3_BO_3_ and CuO/Li_3_BO_3_ Nanocomposites for Visible Light-Driven Photocatalytic Degradation of Dye Pollutant. J. Alloys Compd..

[43] Raha S., Mohanta D., Ahmaruzzaman M. (2021). Novel CuO/Mn_3_O_4_/ZnO Nanocomposite with Superior Photocatalytic Activity for Removal of Rabeprazole from Water. Sci. Rep..

[44] Raizada P., Sudhaik A., Patial S., Hasija V., Khan A.A.P., Singh P., Gautam S., Kaur M., Nguyen V.-H. (2020). Engineering Nanostructures of CuO-Based Photocatalysts for Water Treatment: Current Progress and Future Challenges. Arab. J. Chem..

[45] Muniyalakshmi M., Sethuraman K., Silambarasan D. (2020). Synthesis and Characterization of Graphene Oxide Nanosheets. Mater. Today Proc..

[46] Shah R.M., Yunus R.M., Masdar M.S.M., Minggu L.J., Wong W.Y., Salehmin M.N.I. (2021). High Photoelectrochemical Performance of a P-Type Reduced Graphene Oxide-Copper Oxide/Cu Foil (RGO-CuO/Cu) Photoelectrode Prepared by a One-Pot Hydrothermal Method. Int. J. Energy Res..

[47] Saadi H., Khaldi O., Pina J., Costa T., Seixas de Melo J.S., Vilarinho P., Benzarti Z. (2024). Effect of Co Doping on the Physical Properties and Organic Pollutant Photodegradation Efficiency of ZnO Nanoparticles for Environmental Applications. Nanomaterials.

[48] Tumampos S.B., Ensano B.M.B., Pingul-Ong S.M.B., Ong D.C., Kan C.-C., Yee J.-J., de Luna M.D.G. (2021). Isotherm, Kinetics and Thermodynamics of Cu (II) and Pb (II) Adsorption on Groundwater Treatment Sludge-Derived Manganese Dioxide for Wastewater Treatment Applications. Int. J. Environ. Res. Public Health.

[49] Fang Y., Luo B., Jia Y., Li X., Wang B., Song Q., Kang F., Zhi L. (2012). Renewing Functionalized Graphene as Electrodes for High-Performance Supercapacitors. Adv. Mater..

[50] Wang G., Wang B., Park J., Yang J., Shen X., Yao J. (2009). Synthesis of Enhanced Hydrophilic and Hydrophobic Graphene Oxide Nanosheets by a Solvothermal Method. Carbon.

[51] Thakur S., Karak N. (2012). Green Reduction of Graphene Oxide by Aqueous Phytoextracts. Carbon.

[52] Jose P.P.A., Kala M.S., Kalarikkal N., Thomas S. (2018). Reduced Graphene Oxide Produced by Chemical and Hydrothermal Methods. Mater. Today Proc..

[53] Alves D.C.B., Silva R., Voiry D., Asefa T., Chhowalla M. (2015). Copper Nanoparticles Stabilized by Reduced Graphene Oxide for CO_2_ Reduction Reaction. Mater. Renew. Sustain. Energy.

[54] Vinodha G., Shima P.D., Cindrella L. (2019). Mesoporous Magnetite Nanoparticle-Decorated Graphene Oxide Nanosheets for Efficient Electrochemical Detection of Hydrazine. J. Mater. Sci..

[55] Tran D.-T., Nguyen T.-H., Vu P.-T., Dang V.-Q., Van H.-T. (2024). Cu-ZnO/CdS/RGO Tertiary Heterojunction for Improved Photocatalytic Degradation of Synthetic Dyes Using Visible Light. J. Water Process Eng..

[56] Sagadevan S., Lett J.A., Weldegebrieal G.K., Garg S., Oh W.C., Hamizi N.A., Johan M.R. (2021). Enhanced Photocatalytic Activity of Rgo-Cuo Nanocomposites for the Degradation of Organic Pollutants. Catalysts.

[57] Johra F.T., Lee J.-W., Jung W.-G. (2014). Facile and Safe Graphene Preparation on Solution Based Platform. J. Ind. Eng. Chem..

[58] Zhang Y., Liu J., Zhang Y., Liu J., Duan Y. (2017). Facile Synthesis of Hierarchical Nanocomposites of Aligned Polyaniline Nanorods on Reduced Graphene Oxide Nanosheets for Microwave Absorbing Materials. RSC Adv..

[59] Ranjbar-Azad M., Behpour M. (2021). Facile in Situ Co-Precipitation Synthesis of CuO–NiO/RGO Nanocomposite for Lithium-Ion Battery Anodes. J. Mater. Sci. Mater. Electron..

[60] Verma P., Das T., Kumar P., Das S. (2023). Surface-Passivated RGO@ CuO/6A5N2TU Colloidal Heterostructures for Efficient Removal of Ofloxacin from Contaminated Water through Dual-Mode Complexation: Insights into Kinetics and Adsorption Isotherm Model Study. Appl. Nanosci..

[61] Saxena S., Tyson T.A., Shukla S., Negusse E., Chen H., Bai J. (2011). Investigation of Structural and Electronic Properties of Graphene Oxide. Appl. Phys. Lett..

[62] Zheng F., Xu W.-L., Jin H.-D., Hao X.-T., Ghiggino K.P. (2015). Charge Transfer from Poly(3-Hexylthiophene) to Graphene Oxide and Reduced Graphene Oxide. RSC Adv..

[63] Zhang D., Qin J., Wei D., Yang S., Wang S., Hu C. (2018). Enhancing the CO Preferential Oxidation (CO-PROX) of CuO–CeO_2_/Reduced Graphene Oxide (RGO) by Conductive RGO-Wrapping Based on the Interfacial Charge Transfer. Catal. Lett..

[64] Dhiman P., Rana G., Alshgari R.A., Kumar A., Sharma G., Naushad M., ALOthman Z.A. (2023). Magnetic Ni–Zn Ferrite Anchored on g-C_3_N_4_ as Nano-Photocatalyst for Efficient Photo-Degradation of Doxycycline from Water. Environ. Res..

[65] Yin L., Wang H., Li L., Li H., Chen D., Zhang R. (2019). Microwave-Assisted Preparation of Hierarchical CuO@rGO Nanostructures and Their Enhanced Low-Temperature H_2_S-Sensing Performance. Appl. Surf. Sci..

[66] Huang Q., Kang F., Liu H., Li Q., Xiao X. (2013). Highly Aligned Cu_2_O/CuO/TiO_2_ Core/Shell Nanowire Arrays as Photocathodes for Water Photoelectrolysis. J. Mater. Chem. A.

[67] Carley A.F., Roberts M.W., Santra A.K. (1997). Interaction of Oxygen and Carbon Monoxide with CsAu Surfaces. J. Phys. Chem. B.

[68] Bai H., Guo H., Wang J., Dong Y., Liu B., Xie Z., Guo F., Chen D., Zhang R., Zheng Y. (2021). A Room-Temperature NO_2_ Gas Sensor Based on CuO Nanoflakes Modified with RGO Nanosheets. Sens. Actuators B Chem..

[69] Chiu Y.-H., Chang T.-F.M., Chen C.-Y., Sone M., Hsu Y.-J. (2019). Mechanistic Insights into Photodegradation of Organic Dyes Using Heterostructure Photocatalysts. Catalysts.

[70] Yan X., Qian J., Pei X., Zhou L., Ma R., Zhang M., Du Y., Bai L. (2021). Enhanced Photodegradation of Doxycycline (DOX) in the Sustainable NiFe_2_O_4_/MWCNTs/BiOI System under UV Light Irradiation. Environ. Res..

[71] Wang W., Han Q., Zhu Z., Zhang L., Zhong S., Liu B. (2019). Enhanced Photocatalytic Degradation Performance of Organic Contaminants by Heterojunction Photocatalyst BiVO_4_/TiO_2_/RGO and Its Compatibility on Four Different Tetracycline Antibiotics. Adv. Powder Technol..

[72] Mohammadi M., Rezaee Roknabadi M., Behdani M., Kompany A. (2019). Enhancement of Visible and UV Light Photocatalytic Activity of RGO-TiO_2_ Nanocomposites: The Effect of TiO_2_/Graphene Oxide Weight Ratio. Ceram. Int..

[73] Gao J., Gao Y., Sui Z., Dong Z., Wang S., Zou D. (2018). Hydrothermal Synthesis of BiOBr/FeWO_4_ Composite Photocatalysts and their Photocatalytic Degradation of Doxycycline. J. Alloys Compd..

[74] Deng C., Li S., Wang J., Yang Q., Chen H., Tang F., Yang X. (2025). Fabrication of α-Fe_2_O_3_/TiO_2_ Heterojunction for Photocatalytic Degradation of Doxycycline Hydrochloride. J. Mol. Liq..

[75] Xu B., Zhou J.L., Altaee A., Ahmed M.B., Johir M.A.H., Ren J., Li X. (2020). Improved Photocatalysis of Perfluorooctanoic Acid in Water and Wastewater by Ga_2_O_3_/UV System Assisted by Peroxymonosulfate. Chemosphere.

[76] Zhao K., Lu Z.-H., Zhao P., Kang S.-X., Yang Y.-Y., Yu D.-G. (2021). Modified Tri–Axial Electrospun Functional Core–Shell Nanofibrous Membranes for Natural Photodegradation of Antibiotics. Chem. Eng. J..

[77] Park K., Ali I., Kim J.-O. (2018). Photodegradation of Perfluorooctanoic Acid by Graphene Oxide-Deposited TiO_2_ Nanotube Arrays in Aqueous Phase. J. Environ. Manag..

[78] Prabagar J.S., Yashas S.R., Gurupadayya B., Wantala K., Diganta D.B., Shivaraju H.P. (2022). Degradation of Doxycycline Antibiotics Using Lanthanum Copper Oxide Microspheres under Simulated Sunlight. Environ. Sci. Pollut. Res..

[79] Le T.-H., Tran D.-T., Nghiem L.D. (2023). A Novel Tertiary Magnetic ZnFe_2_O_4_/BiOBr/RGO Nanocomposite Catalyst for Photodegrading Organic Contaminants by Visible Light. Sci. Total Environ..

[80] Ayawei N., Ebelegi A.N., Wankasi D. (2017). Modelling and Interpretation of Adsorption Isotherms. J. Chem..

[81] Langmuir I. (1918). The Adsorption of Gases on Plane Surfaces of Glass, Mica and Platinum. J. Am. Chem. Soc..

[82] Song C., Wu S., Cheng M., Tao P., Shao M., Gao G. (2013). Adsorption Studies of Coconut Shell Carbons Prepared by KOH Activation for Removal of Lead (II) from Aqueous Solutions. Sustainability.

[83] Abdelfatah A.M., El-Maghrabi N., Mahmoud A.E.D., Fawzy M. (2022). Synergetic Effect of Green Synthesized Reduced Graphene Oxide and Nano-Zero Valent Iron Composite for the Removal of Doxycycline Antibiotic from Water. Sci. Rep..

[84] Yu Y., Wu K., Xu W., Chen D., Fang J., Zhu X., Sun J., Liang Y., Hu X., Li R. (2021). Adsorption-Photocatalysis Synergistic Removal of Contaminants under Antibiotic and Cr (VI) Coexistence Environment Using Non-Metal g-C_3_N_4_ Based Nanomaterial Obtained by Supramolecular Self-Assembly Method. J. Hazard. Mater..

[85] Yang K., Ye J., Zhao Y., Ge K., Cao J., Wang S., Zhang Z., Zhang Y., Yang Y. (2021). IO-TiO2/PCN-222 Heterostructure with a Tightly Connected Interface and Its Photocatalytic Activity. ChemistrySelect.

[86] Wu Q., Song Y. (2023). Enhanced Interfacial Charge Migration through Fabrication of p-n Junction in ZnIn_2_S_4_/NiFe_2_O_4_/Biochar Composite for Photocatalytic Doxycycline Hydrochloride Degradation. Chem. Eng. J..

[87] Liu W., Zhou J., Zhou J. (2019). Facile Fabrication of Multi-Walled Carbon Nanotubes (MWCNTs)/α-Bi_2_O_3_ Nanosheets Composite with Enhanced Photocatalytic Activity for Doxycycline Degradation under Visible Light Irradiation. J. Mater. Sci..

[88] Sruthi L., Okla M.K., Janani B., Abdel-Maksoud M.A., Saleh I.A., Abu-Harirah H.A., Khan S.S. (2024). Construction of RGO-Bi_2_Sn_2_O_7_-NiFe_2_O_4_ Nanoheterojunction System for the Enhanced Photodegradation of Doxycycline: A Brief Insight on Degradation Kinetics and Toxicological Evaluation on Allium Cepa. J. Clean. Prod..

[89] Hong P., Li Y., He J., Saeed A., Zhang K., Wang C., Kong L., Liu J. (2020). Rapid Degradation of Aqueous Doxycycline by Surface CoFe_2_O_4_/H_2_O_2_ System: Behaviors, Mechanisms, Pathways and DFT Calculation. Appl. Surf. Sci..

[90] Liu W., Zhou J., Hu Z. (2019). Nano-Sized g-C_3_N_4_ Thin Layer@CeO_2_ Sphere Core-Shell Photocatalyst Combined with H_2_O_2_ to Degrade Doxycycline in Water under Visible Light Irradiation. Sep. Purif. Technol..

[91] Yan M., Hua Y., Zhu F., Gu W., Jiang J., Shen H., Shi W. (2017). Fabrication of Nitrogen Doped Graphene Quantum Dots-BiOI/MnNb_2_O_6_ p-n Junction Photocatalysts with Enhanced Visible Light Efficiency in Photocatalytic Degradation of Antibiotics. Appl. Catal. B.

[92] Klauson D., Šakarašvili M., Pronina N., Krichevskaya M., Kärber E., Mikli V. (2017). Aqueous Photocatalytic Degradation of Selected Micropollutants by Pd-Modified Titanium Dioxide in Three Photoreactor Types. Environ. Technol..

[93] Yu Y., Chen D., Xu W., Fang J., Sun J., Liu Z., Chen Y., Liang Y., Fang Z. (2021). Synergistic Adsorption-Photocatalytic Degradation of Different Antibiotics in Seawater by a Porous g-C_3_N_4_/Calcined-LDH and Its Application in Synthetic Mariculture Wastewater. J. Hazard. Mater..

[94] Swetha S., Abdel-Maksoud M.A., Okla M.K., Janani B., Dawoud T.M., El-Tayeb M.A., Khan S.S. (2023). Triple-Mechanism Driven Fe-Doped *n-n* Hetero-Architecture of Pr_6_O_11_-MoO_3_ Decorated g-C_3_N_4_ for Doxycycline Degradation and Bacterial Photoinactivation. Chem. Eng. J..

[95] Moussa H., Girot E., Mozet K., Alem H., Medjahdi G., Schneider R. (2016). ZnO Rods/Reduced Graphene Oxide Composites Prepared via a Solvothermal Reaction for Efficient Sunlight-Driven Photocatalysis. Appl. Catal. B.

[96] Mahrsi M.I., Chouchene B., Gries T., Carré V., Medjahdi G., Ayari F., Balan L., Schneider R. (2024). 0D/1D CuO-Cu_2_O/ZnO Pn Heterojunction with High Photocatalytic Activity for the Degradation of Dyes and Naproxen. J. Environ. Chem. Eng..

[97] Tatykayev B., Donat F., Alem H., Balan L., Medjahdi G., Uralbekov B., Schneider R. (2017). Synthesis of Core/Shell ZnO/RGO Nanoparticles by Calcination of ZIF-8/RGO Composites and Their Photocatalytic Activity. ACS Omega.

