# Supplementary material for: A Rapid and Complete Photodegradation of Doxycycline Using rGO@CuO Nanocomposite Under Visible and Direct Sunlight: Mechanistic Insights and Real-Time Applicability
Source: Nanomaterials (Basel). 2025 Jun 20;15(13):953. doi: 10.3390/nano15130953 (PMC12251118; doi:10.3390/nano15130953)
Supplement: Supplementary file 1 [file nanomaterials-15-00953-s001.zip › nanomaterials-3642239-supplementary.pdf]

ELECTRONIC SUPPLEMENTARY INFORMATION

A Rapid and Complete Photodegradation of Doxycycline Using rGO@CuO Nanocomposite Under Visible and Direct Sunlight: Mechanistic Insights and Real-time Applicability

Panchraj Verma[1], Subrata Das[1,*] Shubham Raj[1] and Raphaël Schneider [2,]

[1] Applied Chemistry Lab, Department of Chemistry, National Institute of Technology Patna, Bihar 800005

[2] Université de Lorraine, CNRS, LRGP, F-54000 Nancy, France.

* Corresponding author: subrataorgchem@gmail.com

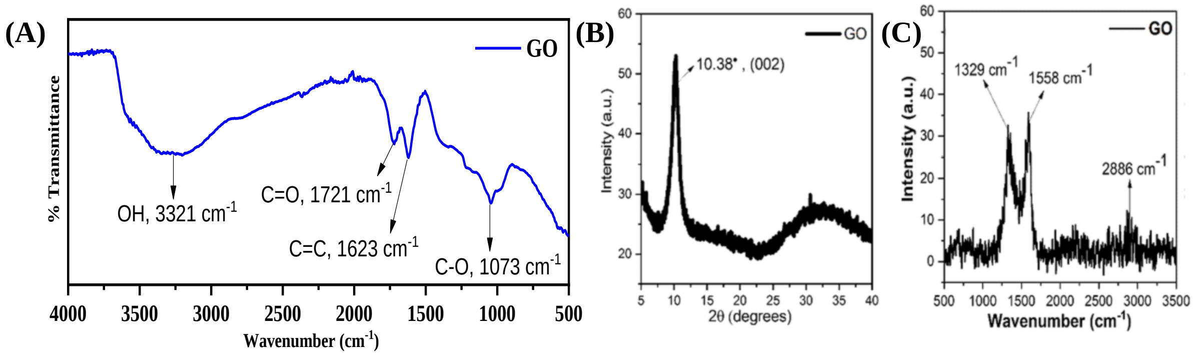

Figure S1. (A) FT-IR spectrum of GO (B) p-XRD of GO (C) Raman analysis of GO.

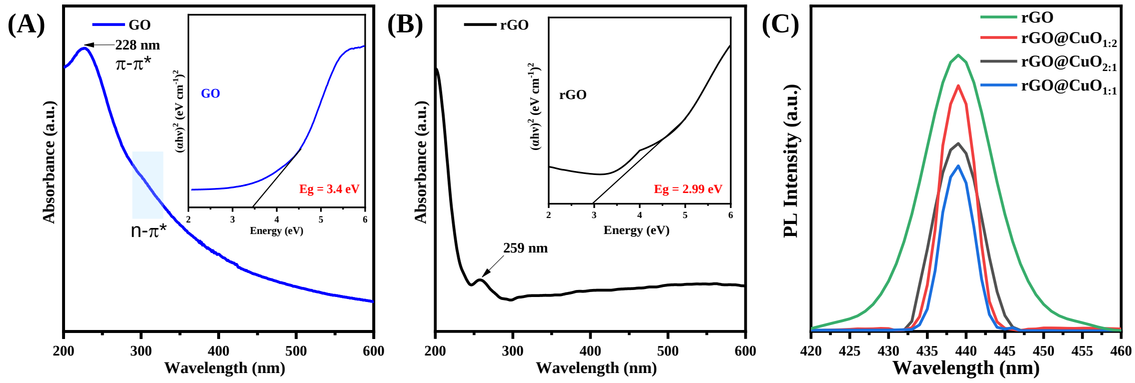

Figure S2. UV-vis spectra of (A) GO and (B) rGO with tauc plot in inset (C) PL analysis of rGO and nanocomposites.

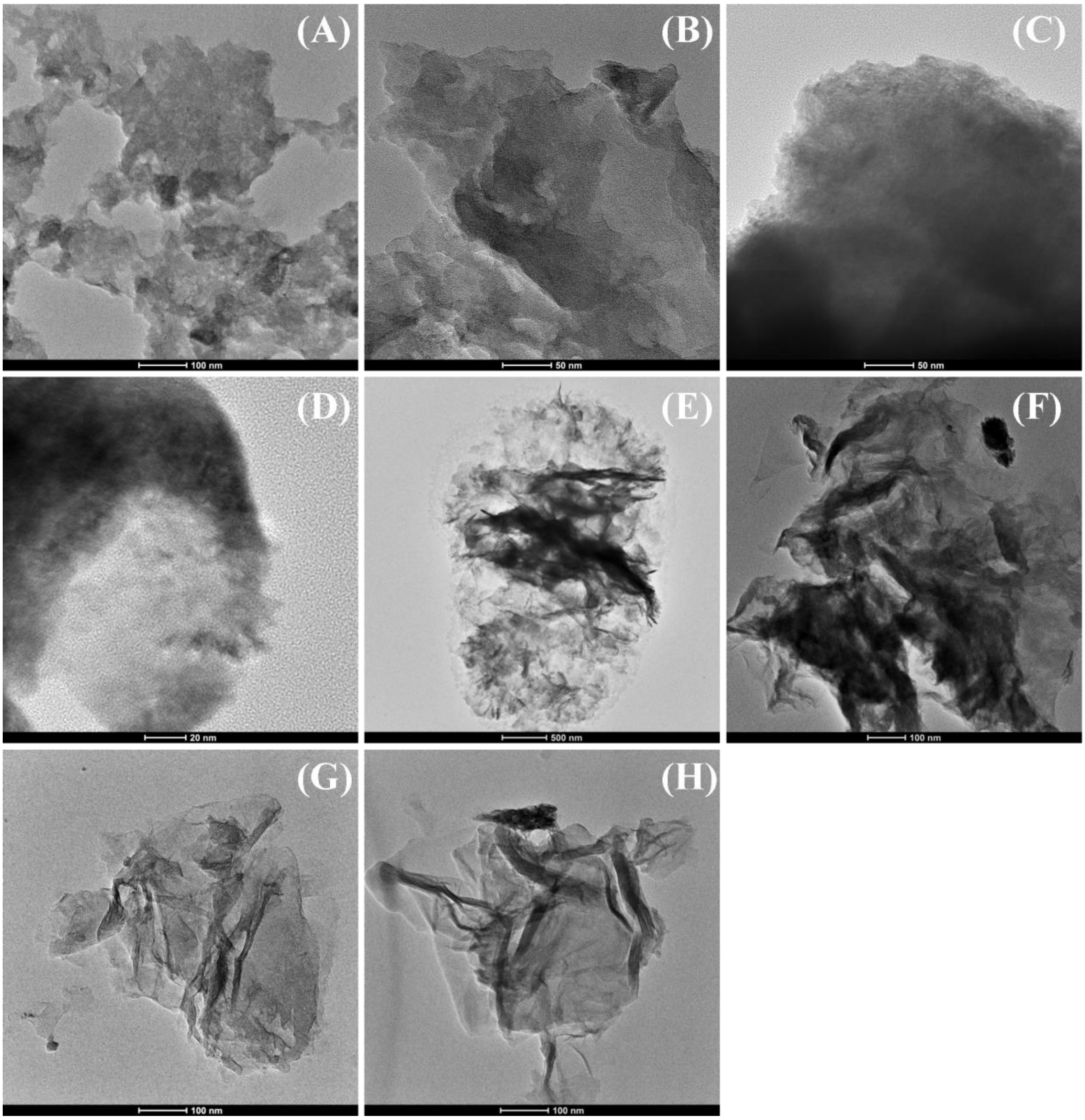

Figure S3. HRTEM images of (A-B) rGO, (C-D) CuO, (E-F) rGO@CuO$_{1:2}$, and (G-H) rGO@CuO$_{2:1}$ at various scales.

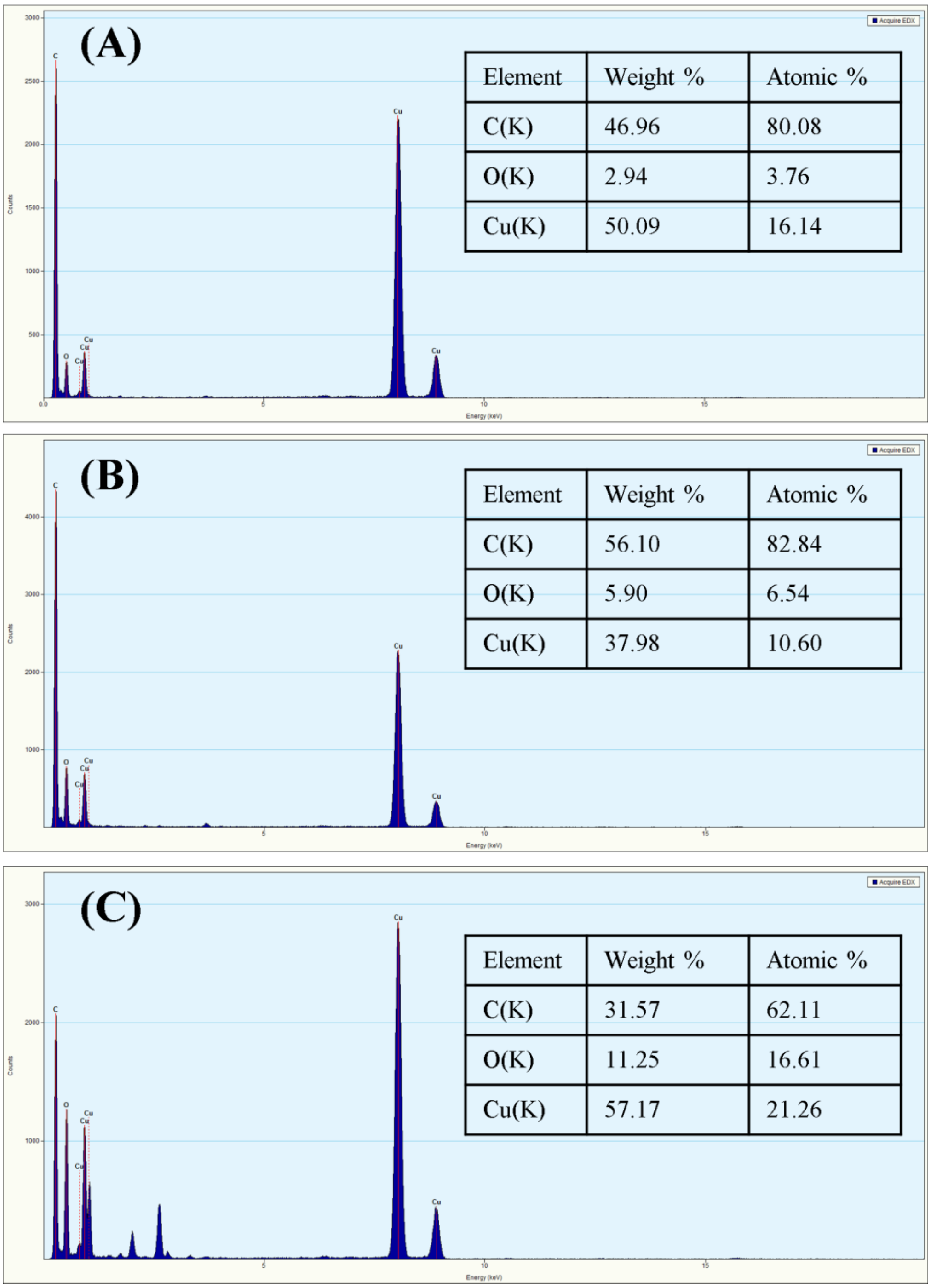

(A)

Element	Weight %	Atomic %
C(K)	46.96	80.08
O(K)	2.94	3.76
Cu(K)	50.09	16.14

(B)

Element	Weight %	Atomic %
C(K)	56.10	82.84
O(K)	5.90	6.54
Cu(K)	37.98	10.60

(C)

Element	Weight %	Atomic %
C(K)	31.57	62.11
O(K)	11.25	16.61
Cu(K)	57.17	21.26

Figure S4. EDX spectra of (A) rGO@$CuO_{1:1}$ (B) rGO@$CuO_{2:1}$ (C) rGO@$CuO_{1:2}$.

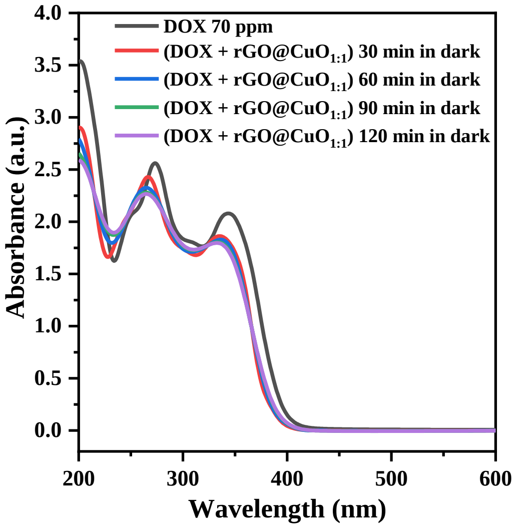

Figure S5. UV-vis absorbance spectra of DOX 70 ppm and DOX 70 ppm + rGO@CuO$_{1:1}$ after keeping them dark for 120 min.

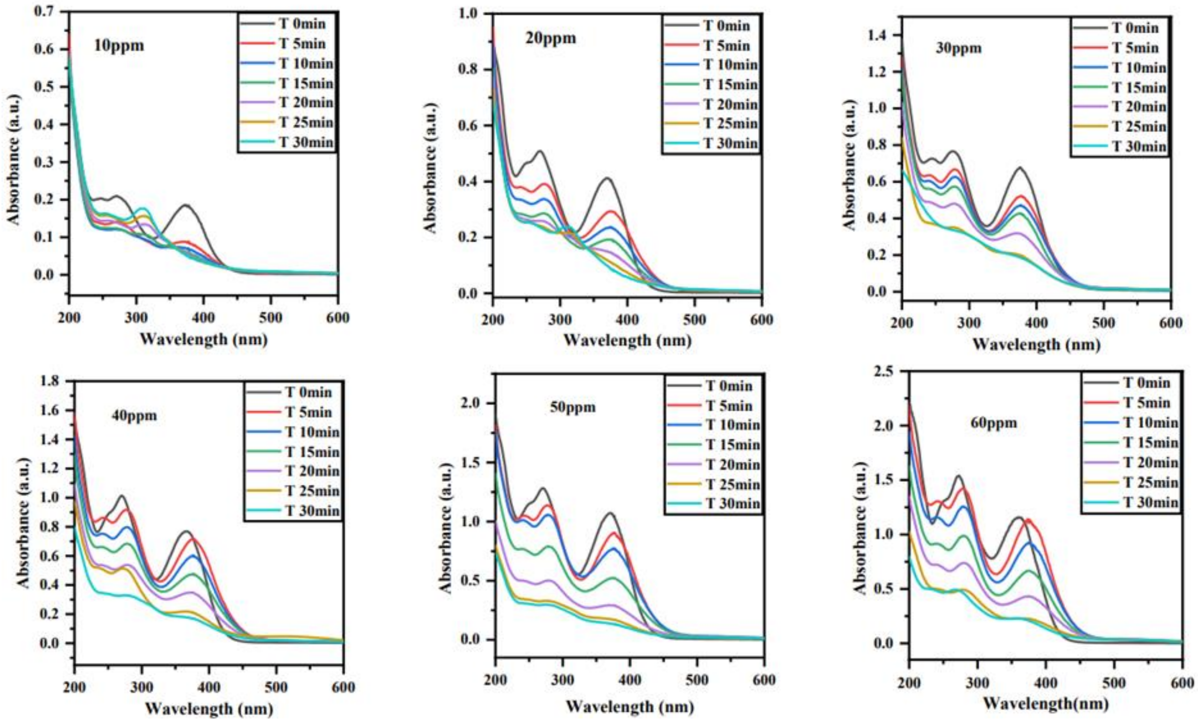

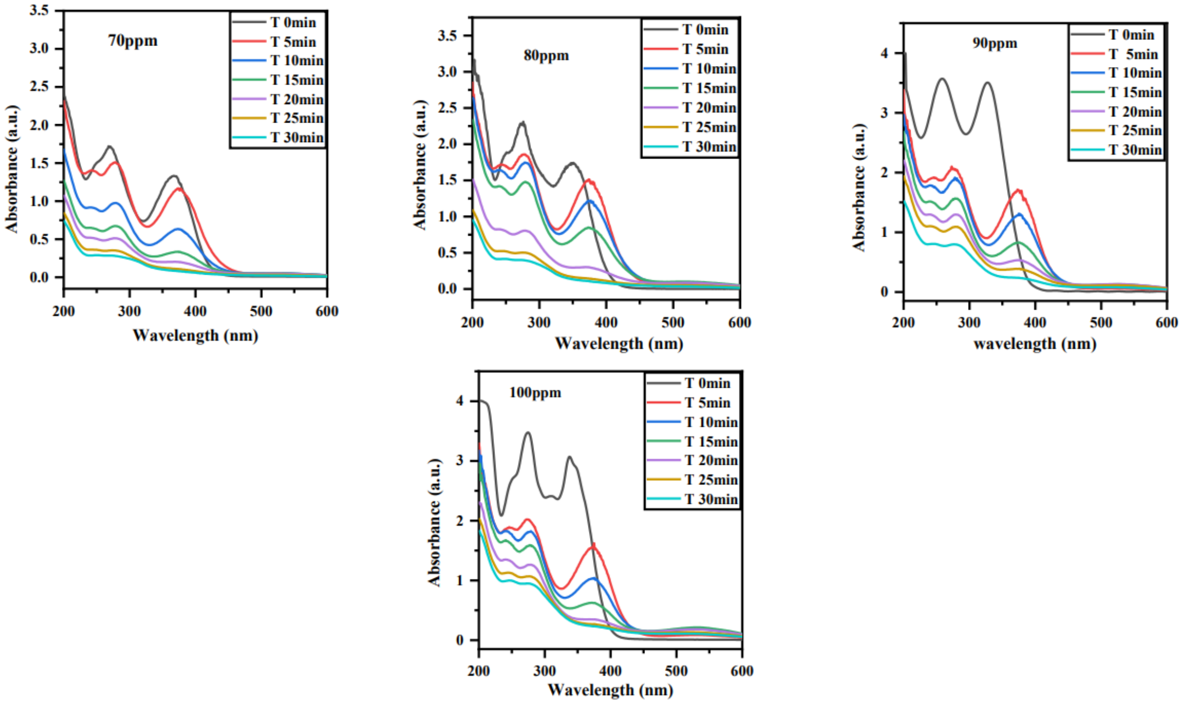

Figure S6. UV–visible absorption spectra for the effect of concentration of DOX on degradation.

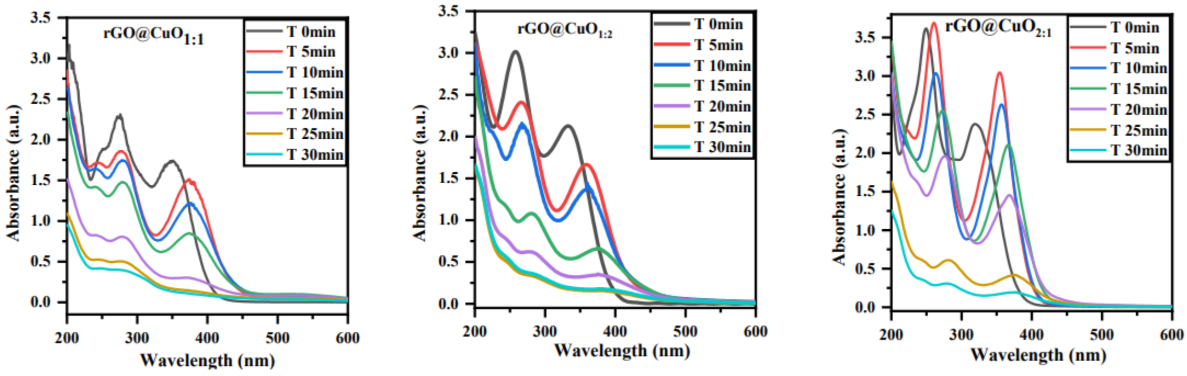

Figure S7. Influence of different compositions of rGO@CuO catalyst on DOX photocatalytic degradation.

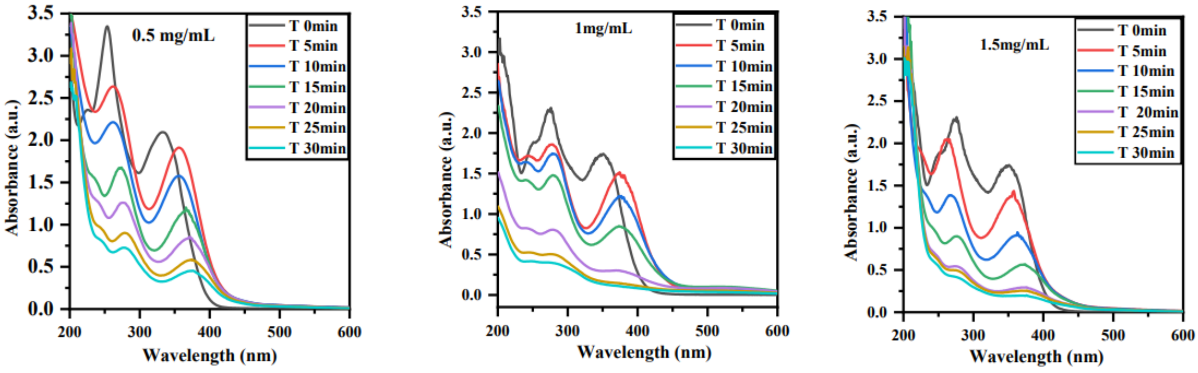

Figure S8. UV-visible spectra of rGO@$CuO_{1:1}$ catalyst loading effect on photocatalytic degradation of DOX.

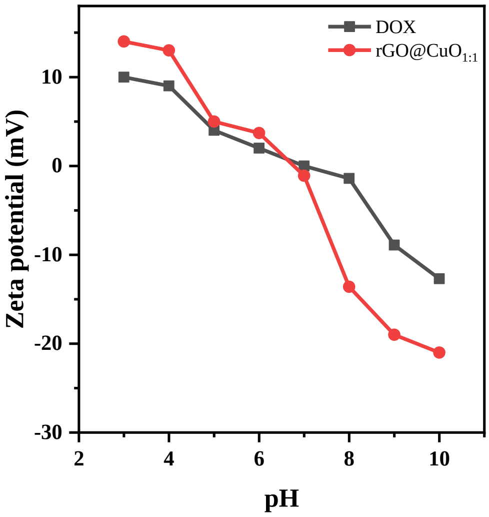

Figure S9. Surface charge of DOX and rGO@$CuO_{1:1}$ catalyst at different pH levels.

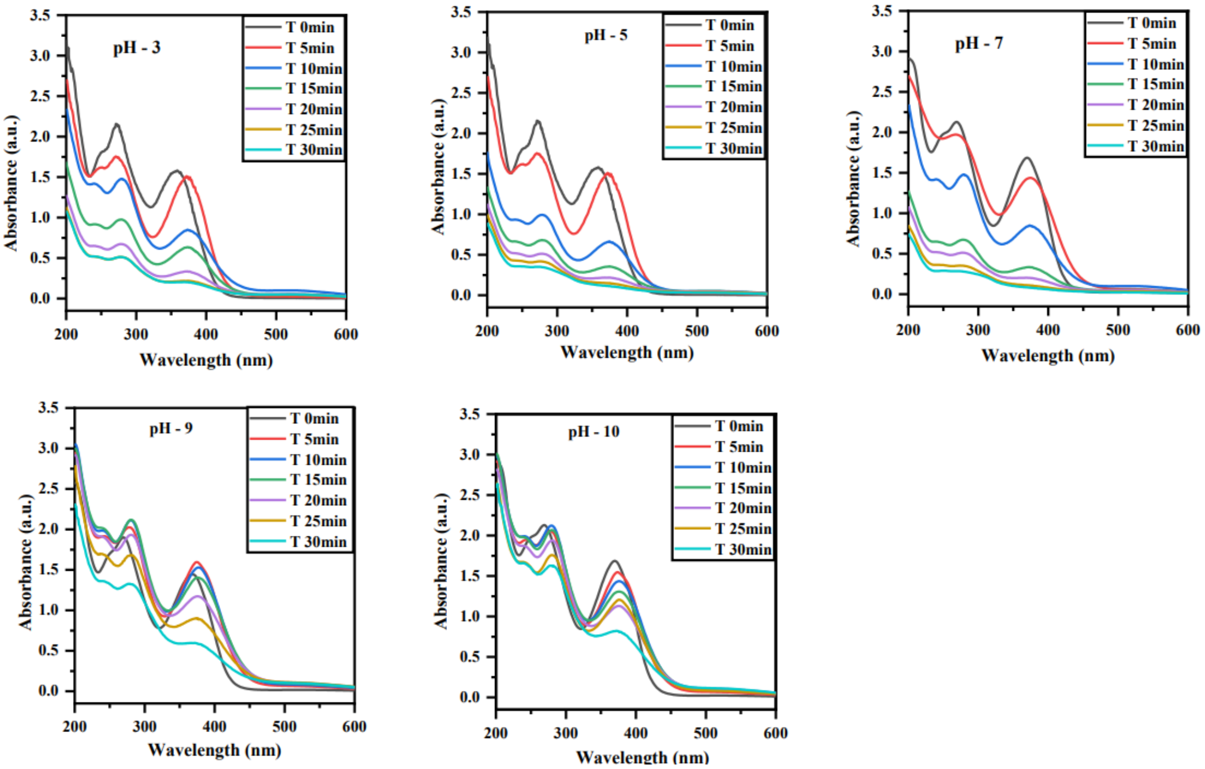

Figure S10. UV-visible absorption spectra of the photocatalytic degradation of DOX by changing pH levels.

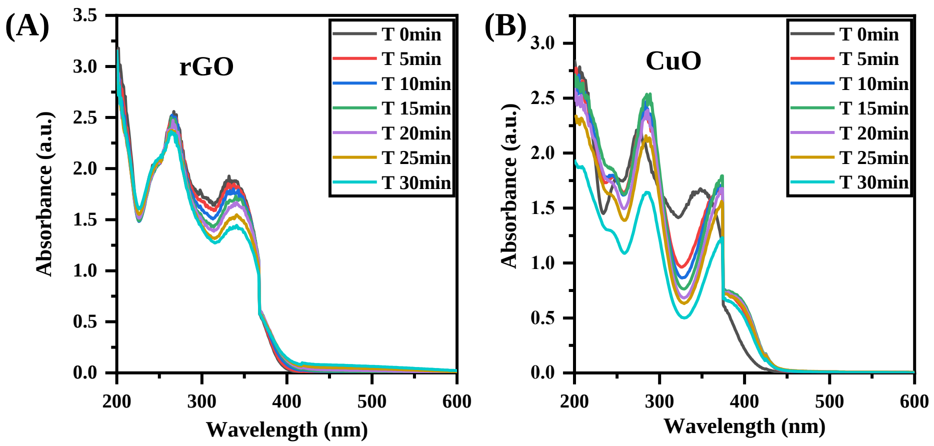

Figure S11. UV-vis absorption spectra of the photocatalytic degradation using the pristine component of the nanocomposite (A) rGO and (B) CuO.

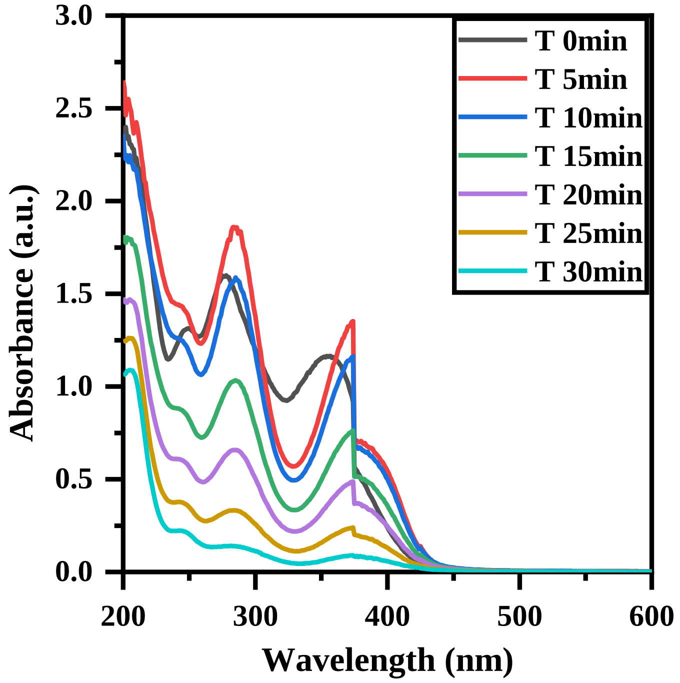

Figure S12. UV-visible absorption spectra for the photodegradation efficiency of rGO@$CuO_{1:1}$ under natural sunlight.

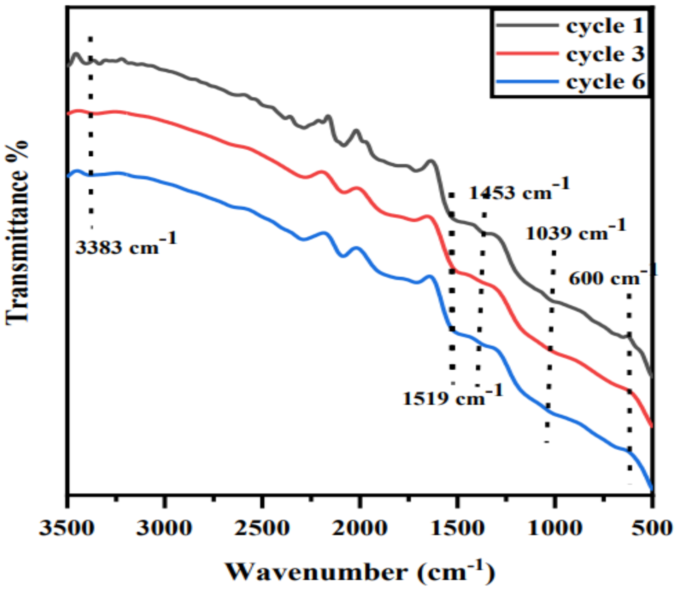

Figure S13. Analysis of FT-IR Spectra of rGO@CuO1:1 catalyst after cycle 1, cycle 3 and cycle 6 of reusability.

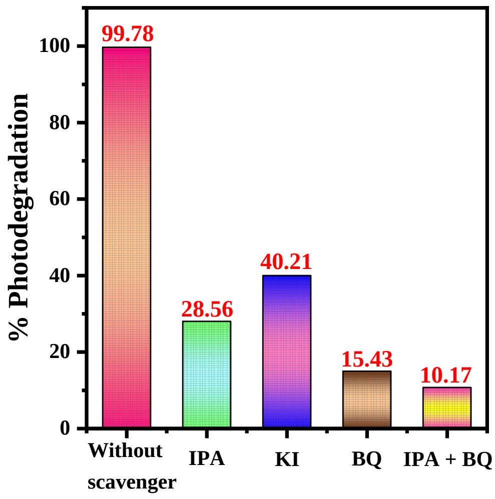

Figure S14. Influence of scavengers on the photocatalytic degradation of DOX using the rGO@CuO1:1 catalyst.

Band edge calculation

The band edge position of CB and VB of CuO was determined using calculated bandgap (E_g ~ 2.82 eV) and Mullikan electronegativity theory as mentioned in eq. (1) and (2).

$$E_{CB} = \chi - E_e - 0.5E_g \quad (1)$$

$$E_{VB} = E_{CB} + E_g \quad (2)$$

Where χ is the absolute electronegativity of CuO (5.81 eV) and E_e is the energy of a free electron on hydrogen scale (4.5 eV). [1] Using eq. (1) estimated value of E_{CB} is -0.1 eV and using it in eq. (2) we get E_{VB} 2.72 eV. From earlier published work, we came to know that the Fermi level of rGO is -0.08 eV. [2]

TOC calculation

The photocatalysts' mineralisation efficiency has been analysed using total organic carbon (TOC) calculation. At optimised parameter TOC of supernatant after photodegradation and 70 ppm DOX (blank) has been analysed. TOC results are mentioned in Table S1 and % mineralisation has been calculated using eq. (3).

Table S1: TOC results before and after photodegradation.

S.N.	Sample	TOC (mg/L)
1.	DOX 70 ppm (blank)	38.2
2.	DOX 70 ppm after photodegradation	12.2

$$\% \text{ mineralisation} = \frac{\{TOC\ (blank) - TOC\ (after\ photodegradation)\}}{TOC\ (blank)}\ x\ 100 \quad (3)$$

References

[1]. Mahrsi, M. I.; Chouchene, B.; Gries, T.; Carré, V.; Medjahdi, G.; Ayari, F.; Balan L.; Schneider, R. 0D/1D CuO-Cu2O/ZnO pn heterojunction with high photocatalytic activity for the degradation of dyes and Naproxen. *J. Environ. Chem. Eng*. **2024**, *12*, 113072. https://doi.org/10.1016/j.jece.2024.113072

[2]. Tatykayev, B.; Donat, F.; Alem, H.; Balan, L.; Medjahdi, G.; Uralbekov, B.; Schneider, R. Synthesis of core/shell ZnO/rGO nanoparticles by calcination of ZIF-8/rGO composites and their photocatalytic activity. *ACS Omega* **2017**, *2*, 4946-4954. https://doi.org/10.1021/acsomega.7b00673
